# TGFβ signalling acts as a molecular brake of myoblast fusion

**DOI:** 10.1038/s41467-020-20290-1

**Published:** 2021-02-02

**Authors:** Julie Melendez, Daniel Sieiro, David Salgado, Valérie Morin, Marie-Julie Dejardin, Chan Zhou, Alan C. Mullen, Christophe Marcelle

**Affiliations:** 1 Institut NeuroMyoGène (INMG), University Claude Bernard Lyon1, CNRS UMR 5310, INSERM U1217, Lyon, France; 2 Australian Regenerative Medicine Institute (ARMI), Monash University, Clayton, VIC Australia; 3 Gastrointestinal Unit, Massachusetts General Hospital, Boston, MA 02114 USA; 4 Harvard Stem Cell Institute, Cambridge, MA 02138 USA; 5 Present Address: Plexus Ventures LLC, Boston, MA USA; 6 Present Address: Marseille Medical Genetics (MMG), Aix Marseille University, INSERM U1251, Marseille, France

**Keywords:** Musculoskeletal development, Muscle stem cells

## Abstract

Fusion of nascent myoblasts to pre-existing myofibres is critical for skeletal muscle growth and repair. The vast majority of molecules known to regulate myoblast fusion are necessary in this process. Here, we uncover, through high-throughput in vitro assays and in vivo studies in the chicken embryo, that TGFβ (SMAD2/3-dependent) signalling acts specifically and uniquely as a molecular brake on muscle fusion. While constitutive activation of the pathway arrests fusion, its inhibition leads to a striking over-fusion phenotype. This dynamic control of TGFβ signalling in the embryonic muscle relies on a receptor complementation mechanism, prompted by the merging of myoblasts with myofibres, each carrying one component of the heterodimer receptor complex. The competence of myofibres to fuse is likely restored through endocytic degradation of activated receptors. Altogether, this study shows that muscle fusion relies on TGFβ signalling to regulate its pace.

## Abstract (web summary)

Fusion of myoblasts is essential for muscle development and repair, but the molecular mechanism underlying this process remains unclear. Here, the authors show, using chicken embryos as a model, that TGFβ signalling inhibits fusion via a receptor complementation mechanism, and indicate the involvement of endocytic degradation of activated receptors in modulation of this process.

## Introduction

Myoblast fusion occurs after muscle specification and early differentiation, themselves regulated by the myogenic regulatory factors (MRFs) MYF5, MYOD and MYOG^1–3^. While MRF function is necessary for myoblast fusion^4^, terminal differentiation, including muscle contraction, can occur even if fusion is disrupted^5–7^. Work in *Drosophila* identified a number of molecules, mainly implicated in actin regulation, required for muscle fusion in this organism. A major advance was the demonstration that their homologues play similar function during vertebrate myogenesis^2,3,8^. Together with vertebrate-specific muscle fusion genes (e.g. Myomaker, Myomixer, JAM2-3) they constitute the fusion machinery necessary for the fusion of myoblasts to myofibres. A recent analysis of myoblast fusion during chicken embryonic development showed that the various myogenic populations that co-exist in the muscle masses display an unexpected variety of fusion behaviours, suggesting that additional mechanisms must exert temporal and spatial control on the fusion machinery, modulating whether progenitors that are competent to fuse do so and at what pace^9^. The molecular underpinning of such control over fusion is unknown.

Here, we uncovered that TGFβ signalling acts as a molecular brake on muscle fusion. Inhibition of the pathway results in a boost in the pace of myoblast fusion, while its constitutive activation leads to a near complete arrest of fusion. The cellular mechanism whereby TGFβ inhibits fusion relies on receptor complementation, triggered by the merging of myoblasts with myofibres, each carrying a component of the heterodimer receptor complex. The competence to fuse is likely restored through endocytic degradation of the activated receptor.

## Results

### Identification of novel regulators of myoblast fusion

**Fig. 1:**
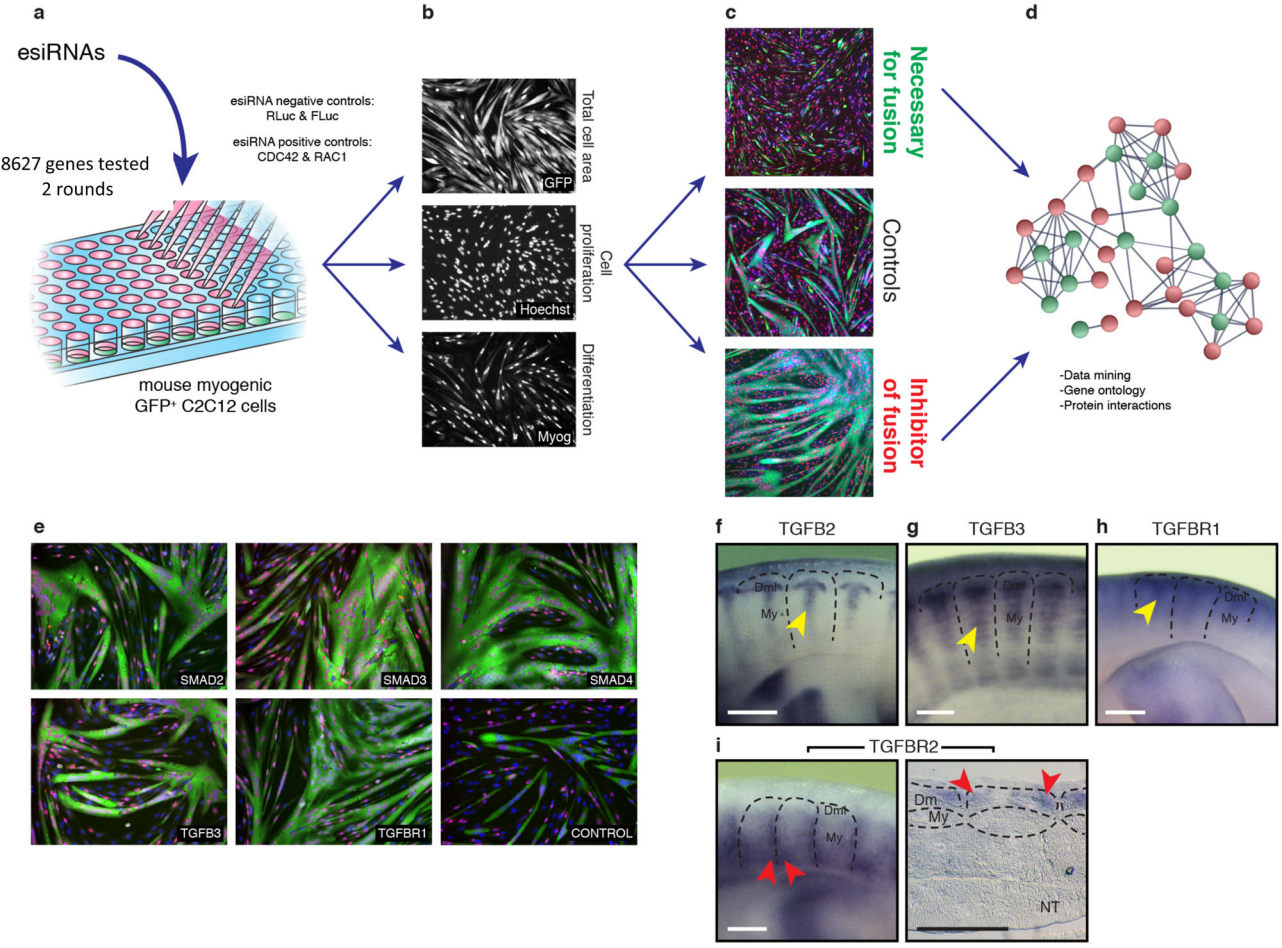
TGFβ signalling inhibits C2C12 fusion, and is expressed in the chicken embryo. **a** EsiRNAs corresponding to 8627 distinct mouse genes were transfected into a (GFP-positive) C2C12 mouse muscle cell line to identify novel molecules regulating myoblast fusion. **b** After differentiation, the area of myofibres (GFP staining), the nuclei count (Hoechst) and myogenin expression were evaluated with an image analysis program. **c** Quantification of the number of nuclei per myofibre identified a number of genes that are either necessary or inhibit myoblast fusion in vitro, compared to controls. **d** Gene ontology (Panther) and protein interaction (String) analyses showed that the TGFβ pathway is over-represented within the inhibitors of fusion. **e** Loss of function of ligands, receptors or effectors of the TGFβ pathway led to a strong increase of C2C12 fusion, compared to controls. **f**–**h** Lateral view of whole-mount (WM) in situ hybridization of chicken embryos at E5.5. The secreted ligands TGFB2 and TGFB3, as well as TGFBR1 are expressed in the myotome of the chicken embryo (yellow arrowheads, **f**–**h**). **i** In contrast, TGFBR2 is expressed in epithelial cells located at the anterior and posterior borders of the dermomyotome (shown in WM and longitudinal section of chicken embryos at E5.5, red arrowheads). Dm: dermomyotome, My: myotome, NT: neural tube. Scale bars **f**–**i**: 0.5 mm.

To uncover those mechanisms, we performed an in vitro esiRNA (endonuclease-cleaved siRNA) screen on the mouse myogenic cell line C2C12. The esiRNA library represented about 9000 independent genes (i.e. one-third of the mouse genome). This identified a large array of genes that either activate or inhibit C2C12 fusion, with little or no effect on differentiation and proliferation (Fig. 1a–c, Supplementary Fig. 1 and Supplementary Tables 1, 2). Validating the approach, we observed that molecules previously known to be necessary for myoblast fusion in vertebrates and invertebrates were identified through the screen (Supplementary Table 3). These data constitute a valuable resource for genes tested for fusion, proliferation and myogenic differentiation.

Gene ontology analysis software (pantherdb.org) indicated that the TGFβ signalling pathway was over-represented within the group of molecules inhibiting the fusion of C2C12 cells (Fig. 1d, Supplementary Fig. 1 and Supplementary Table 4). Since the vast majority of molecules modulating fusion were found to be necessary for fusion, we sought to examine the possibility that the TGFβ signalling pathway may display an inhibitory function in this process in vivo. The TGFβ family is a large group of ligands that signal via heterotetrameric complexes of two type I and two type II receptors, which mediate their activity intra-cellularly via R-SMADs (Supplementary Fig. 2). TGFBs, activins, myostatin and nodal induce the phosphorylation of R-SMAD2 or 3, while BMPs mediate their activity through R-SMAD1, 5 or 8. All R-SMADs bind to SMAD4, an essential component for the generation of SMAD transcriptional responses^10–12^. Previous studies have demonstrated a role for some TGFβ ligands in myogenesis. Myostatin (MSTN) is best known for the dramatic increase of muscle mass, first identified in cattle, resulting from its loss-of-function. This phenotype is due to its crucial role in the Akt/mTOR/p70S6K protein synthesis pathway in adult muscle fibres^13–15^. MSTN also plays an important function on the early steps of myogenesis in the embryo, promoting the premature activation of MYOD expression in embryonic (named resident) muscle progenitors, thereby leading to the gradual exhaustion of the muscle stem cell pool^16^. In contrast, BMP strongly inhibits the activation of MYOD expression in embryonic muscle progenitors^17^. Relevant to the present study, it was observed that TGFB and SMAD3 repress MYOD expression in vitro^18–20^. Altogether, these data suggest that different members of the TGFβ superfamily have distinct, and sometimes opposite, functions on myogenesis, suggesting that their roles are cell context-dependent. Importantly, changes in the early stages of myogenesis impact all subsequent steps of the myogenic program and it is thus not surprising that a failure to activate early myogenesis would lead to the marked decrease in myoblast fusion that was reported in some of those studies. However, whether TGFBs and their downstream effectors have a specific role during myoblast fusion in vivo is unknown.

In the screen, in addition to bona fide components of the TGFβ signalling pathway (TGFβ ligands -TGFBs-, receptor -TGFBR1- and SMAD effectors), we also identified positive (DPT, MMP14, RUNX1, SCUBE3) and negative (TGIF1) regulators of the pathway that promoted (TGIF1) or inhibited (all others) the fusion of C2C12 cells (Fig. 1e and Supplementary Table 4). Significantly, SMAD4, SMAD3, TGFB2 and TGFBR1 were among the 10% strongest inhibitors of fusion we identified in this screen. It is notable that none of the tested genes significantly influenced proliferation. While they displayed a mild inhibitory effect on myogenic differentiation (i.e. on Myogenin expression) as shown previously^18–20^, the effect on fusion was in the majority of cases stronger than on differentiation, suggesting that their inhibitory activity on fusion was not solely due to their activity on upstream events of the myogenic program. Importantly, among the tested molecules, we found that the members of the BMP signalling pathway (BMP1, BMP4, BMP7, BMP8, GDF5, BMPR1A), Inhibins (INHBA, INHBC, INHBE), the type 2 receptors for MSTN (ACVR2A, ACVR2B) and the inhibitor of MSTN, Follistatin (FST) did not influence C2C12 fusion (Supplementary Table 4). These data suggest that TGFBs (but neither the SMAD1/5/8-dependent BMPs, nor the SMAD2/3-dependent ligands MSTN or INHBs) act as inhibitors of myoblast fusion in vitro.

### SMAD2/3 TGFβ signalling acts as a molecular brake of myoblast fusion

We determined whether the TGFβ family members we identified were expressed at the time and place where we know that muscle fusion is taking place during embryonic development^9^. In the chicken embryo, trunk and limb muscle fibres initiate fusion at E4.5 (Stage HH25^21^). While the R-SMADs SMAD2 and SMAD3 are widely expressed in all embryonic tissues^22^, we found that the ligands TGFB2 and TGFB3 were specifically expressed in the myotome of somites from E4.5 (Fig. 1f, g). At E4.5, the myotome is composed of mononucleated, terminally differentiated primitive myofibres, named myocytes. Myocytes are attached at the anterior and posterior borders of each somite and they express the embryonic form of MyHC (myosin heavy chain), recognized by the MF20 antibody (*24*–*26*; Supplementary Fig. 3). TGFB2 and 3 signal through TGFBR1 and TGFBR2^10^. TGFBR1 was expressed in the myotome of somites at E4.5 (Fig. 1h). Myocytes, therefore, express TGFB2 and 3 and TGFBR1. Remarkably, we observed that TGFBR2 was expressed at the anterior and posterior epithelial borders of each somite, but not in the medial border of the dermomyotome (DML) or in the myotome (Fig. 1i). This is important, since we recently demonstrated that myocytes derived from the DML exclusively fuse to progenitors originating from the anterior and posterior borders of somites^9^ (Supplementary Fig. 3).

To address the role of TGFβ signalling in myogenic differentiation and myoblast fusion of trunk muscles, we activated the pathway in the DML, using the in vivo electroporation technique^23–25^. The DML comprises Pax7/Pax3-positive epithelial progenitor muscle cells that generate, over many days and in concert with the other dermomyotome borders, the myocytes that populate the myotome (Supplementary Fig. 4). To enter the myogenic program, these progenitors react to signals from their environment, sequentially activating MRFs and an entire array of genes specific to mature skeletal muscles (e.g. MLC, MyHC, MCK, Dystrophin, etc.). We have taken two distinct approaches to address the role of TGFβ signalling in the DML. First, we have electroporated TGF-activating and -inhibiting constructs in DML cells of E2.5 embryos using an ubiquitous (CAGGS) promoter. We have previously shown that this promoter drives robust expression of genes under its control in DML cells in as little as three hours (*24;* Supplementary Fig. 4,d). This is before myotome formation is initiated^25^ and this should result in the overexpression of the constructs in the progenitor population and their progeny during the entire time of the experiment. We tested the role of a WT form of TGFBR2 and of SMAD3, to activate the pathway, while a DN form of TGFBR1 was used to inhibit the pathway. Since TGFBR1 dimerizes with the type II receptors TGFBR2, ACVR2A and ACVR2B (all of which acting through R-SMAD2 or 3^10^), a DN form of TGFBR1 is likely to inhibit all three heterotetrameric complexes. The formation of myocytes derived from these electroporated progenitors was evaluated two days later (E4.5). This experiment thus aims at testing whether the activation or inhibition of TGFβ signalling in early muscle progenitor cells alters their potential to undergo myogenic differentiation in vivo.

Second, we activated the pathway, using a myosin light chain (MLC) promoter^26^. MLC is a gene activated later in the myogenic program and accordingly, we found that its activity was initiated in myogenin expressing, terminally differentiating myogenic cells (Supplementary Fig. 5). The MLC promoter is thus appropriate to address gene function in later stages of the myogenic program, and in the present study, during myoblast fusion. For this series of experiments, DML cells were electroporated at E2.5 and the number of nuclei per fibre was evaluated 3 days later at E5.5.

**Fig. 2:**
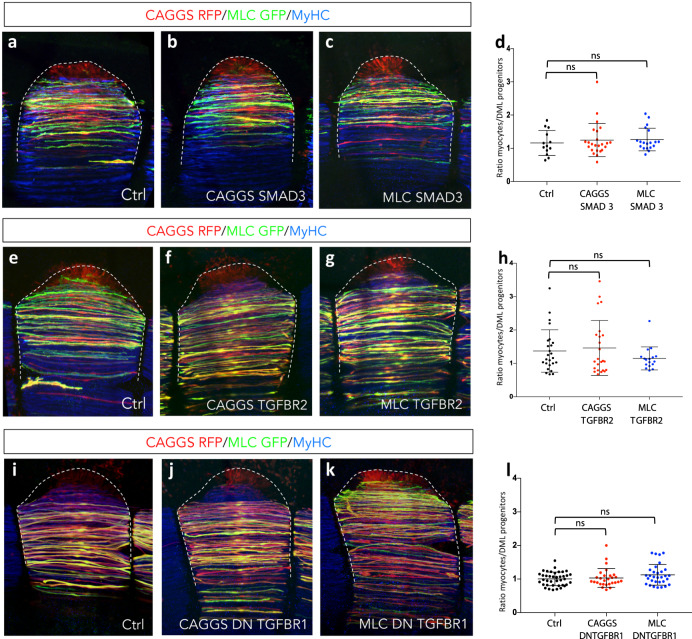
SMAD2/3-dependent TGFβ signalling does not regulate myogenic differentiation in vivo. **a**–**c**, **e**–**g**, **i**–**k** Dorsal views of confocal stacks of somites observed at E4.5. To test myogenic differentiation, they were electroporated in the DML at E2.5 with the ubiquitous (CAGGS) promoter (**b**, **f**, **j**) or the muscle specific MLC promoter (**c**, **g**, **k**) driving expression of SMAD3 (**b**, **c**), TGFBR2 (**f**, **g**) and DN TGFBR1 (**j**, **k**). (CAGGS empty vector as control). They were also co-electroporated with two plasmids, CAGGS RFP (red) and MLC GFP (green) that label all electroporated cells or only myocytes, respectively; in blue, MyHC staining. **d**, **h**, **l** Scatter plots graphs showing the ratio of myocytes generated from the electroporated DML progenitors in each conditions. Statistical analyses: CAGGS SMAD3: $\bar x$: 1.25; *n* = 24; CAGGS SMAD3 vs Ctrl, Mann–Whitney non-parametric test: *P*-value: 0.71; MLC SMAD3: $\bar x$: 1.27; n = 19; *P*-value: 0.32; Ctrl: $\bar x$: 1.16; *n* = 13. CAGGS TGFBR2: $\bar x$: 1.46; *n* = 24; *P*-values: 0.99; MLC TGFBR2: $\bar x$: 1.15; *n* = 11; *P*-value: 0.38; Ctrl: $\bar x$: 1.37; *n* = 25. CAGGS DN TGFBR1: $\bar x$: 1.03; *n* = 28; *P*-value: 0.86; MLC DN TGFBR1: $\bar x$: 1.13; *n* = 3; *P*-value: 0.19; Ctrl: $\bar x$: 1.01; *n* = 39. In **d**, **h**, **l** means and standard deviation are indicated; ns: non statistically significant difference. Source data are provided (see ‘Data availability’).

Two days after electroporation of CAGGS driving TGFBR2, SMAD3 and DN TGFBR1, we quantified the number of differentiated cells (myocytes) generated from electroporated DML progenitors. We observed that neither the activation (TGFBR2 and SMAD3) nor the inhibition (DN TGFBR1) of the pathway had a significant effect on the formation of myocytes, compared to controls (Fig. 2a–l). As a second control, we used the same three constructs, driven by a MLC promoter. As expected, they did not have a significant effect on the emergence of myocytes either. All experimental constructs, however, modulated the fusion of myofibres (TGFBR2 and SMAD3, inhibition; DN TGFBR1, activation of fusion; see below). These data indicate that, contrary to what has been reported in vitro (*19*–*21* and our data above), SMAD3-dependent TGFβ signalling does not control the myogenic differentiation of muscle progenitors in early chicken embryos.

**Fig. 3:**
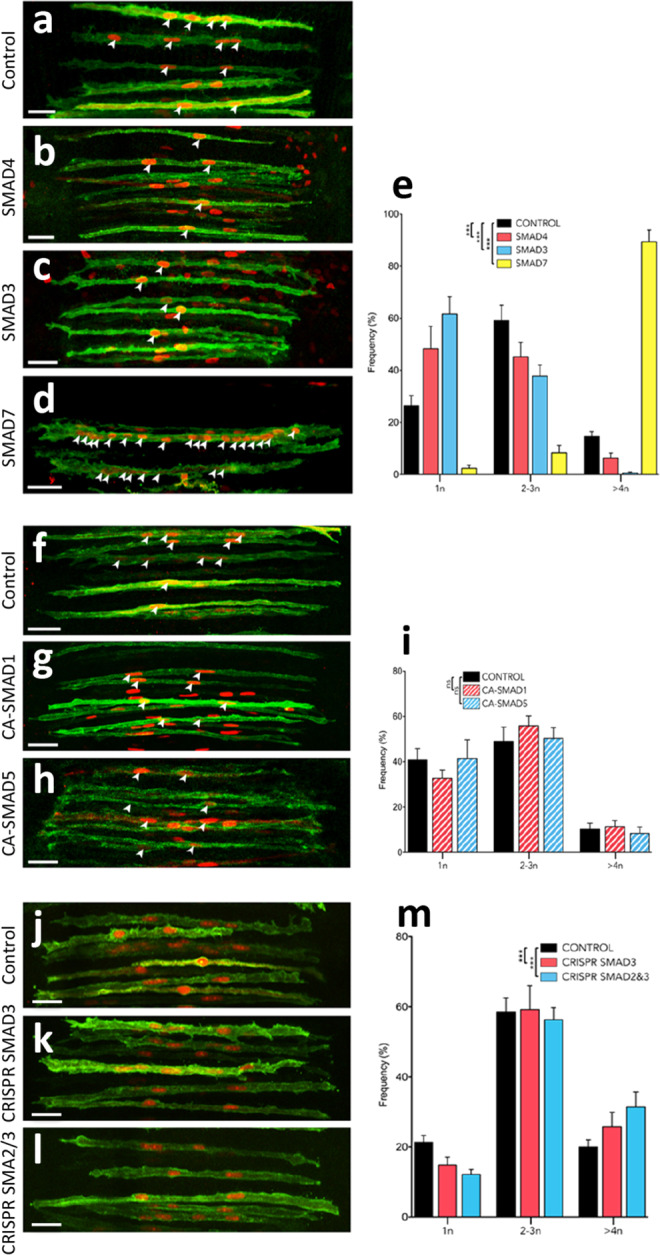
SMAD2/3-dependent TGFβ signalling regulates myoblast fusion in vivo. **a**–**d**, **f**–**h**, **i**–**l** Dorsal views of confocal stacks of somites observed at E5.5. To test myoblast fusion, they were electroporated in the DML at E2.5 with the following: **a**–**d** electroporation of MLC promoter driving expression of the indicated genes, together with membranal GFP (green) and nuclear mCherry (red) allowing the quantification of nuclei per myocyte. **e** Column graph for **a**–**d** showing the frequency distribution of electroporated myocytes distributed in those that contained one, two to three, and four or more nuclei, relative to its control (in %). **f**–**h** Electroporation of the BMP-related SMADs together with membranal GFP (green) and nuclear mCherry (red). **i** Column graph for **f**–**h** showing the frequency distribution of fusion events relative to controls (in %). **j**–**l** Electroporation of a CRISPR/Cas9 construct targeting the chicken SMAD3 sequence alone (**k**) or together with a CRISPR/Cas9 construct targeting the chicken SMAD2 sequence (**l**) together with membranal GFP (green) and nuclear mCherry (red). **m** Column graph for **j**–**l** showing the frequency distribution of fusion events relative to controls (in %). Arrowheads point to cell nuclei in selected fibres. Statistical analyses: SMAD4: $\bar x$: 1.72; *n* = 7; Ctrl: $\bar x$: 2.29; *n* = 19; *P*-value < 0.0001. SMAD3: $\bar x$: 1.48; *n* = 15; Ctrl: $\bar x$: 2.29; *n* = 19; *P*-value < 0.0001; SMAD7: $\bar x$: 7.55; *n* = 8; Ctrl: $\bar x$: 2.29; *n* = 19; *P*-value < 0.0001; CA SMAD1: $\bar x$: 2.11; *n* = 11; Ctrl: $\bar x$: 1.98; *n* = 7; *P*-value: 0.16; CA SMAD5: $\bar x$: 1.95; *n* = 10; Ctrl: $\bar x$: 1.98; *n* = 7; *P*-value: 0.97; CRISPR SMAD3: $\bar x$: 2.86; *n* = 24; Ctrl: $\bar x$: 2.57; *n* = 29; *P*-value = 0.0002; CRISPR SMAD2/3: $\bar x$: 2.95; *n* = 47; Ctrl: $\bar x$: 2.57; *n* = 36; *P*-value < 0.0001; Error bars in **e**, **i**, **m**: SEM. Scale bars: 50 μm. Source data are provided (see ‘Data availability’).

**Fig. 4:**
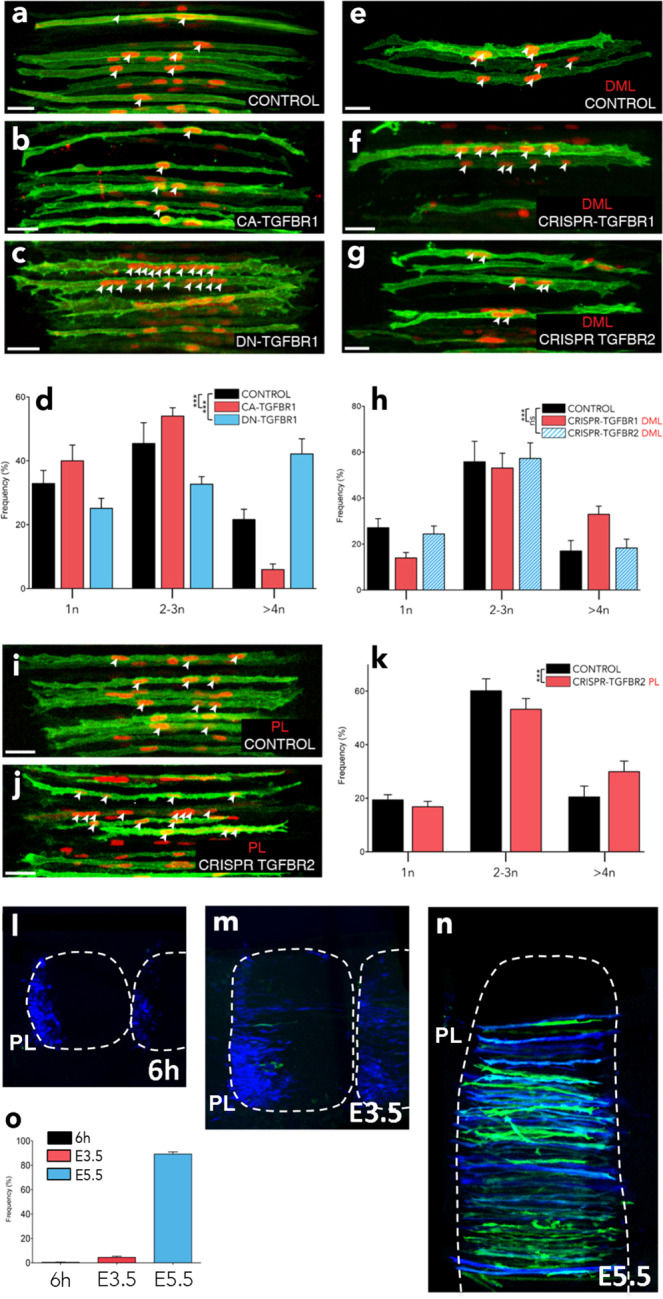
TGFBR1 in the DML and TGFBR2 in the posterior somite are necessary for fusion. **a**–**c** DML-derived myocytes electroporated with MLC promoter driving expression of constitutively active (CA, **b**) and dominant-negative (DN, **c**) TGFBR1 variants, together with membrane GFP (green) and nuclear mCherry (red). **d** Column graph for **a**–**c** showing the population of electroporated myocytes containing the indicated number of nuclei relative to their controls (in %). **e**–**g** DML-derived myocytes electroporated with a CRISPR/Cas9 construct targeting the chicken TGFBR1 or TGFBR2 sequences together with membrane GFP (green) and nuclear mCherry (red). **h** Column graph for **e**–**g** showing the population of electroporated myocytes containing the indicated number of nuclei relative to their controls (in %). Solid colour columns indicate overall significant difference with controls. Striped colour columns indicate non-significant difference with controls. **i**, **j** Myocytes electroporated in the posterior border of the dermomyotome with a CRISPR/Cas9 construct targeting the chicken TGFBR2 sequence together with membrane GFP (green) and nuclear mCherry (red). **k** Column graph for i-j showing the population of electroporated myocytes containing the indicated number of nuclei relative to their controls (in %). Embryos in **a**–**c**, **e**–**g**, **i**, **j** and **l**–**n** were fixed at E5.5 and stained against GFP and RFP antibodies. **l**–**n** Somites electroporated in the posterior border (PL) with cytoplasmic BFP (in blue) as electroporation marker and the TGF β reporter we identified, upstream of eGFP (in green). Somites were analysed 6 h (**l**), one day (**m**) and 3 days (*n*) after electroporation. **o** Column graph for **l**–**n** showing the percentage of electroporated cells that express the reporter. Statistical analyses: CA TGFBR1: $\bar x$: 1.88; *n* = 6; Ctrl: $\bar x$: 2.46; *n* = 13; *P*-value =0.0004; DN TGFBR1: $\bar x$: 3.56; *n* = 9; Ctrl: $\bar x$: 2.46; *n* = 13; *P*-value < 0.0001; CRISPR DML TGFBR1: $\bar x$: 3.02; *n* = 21; Ctrl: $\bar x$: 2.43; *n* = 14; *P*-value < 0.0001; CRISPR DML TGFBR2: $\bar x$: 2.47; *n* = 21; Ctrl: $\bar x$: 2.43; *n* = 14; *P*-value: 0.97; CRISPR PB TGFBR2: $\bar x$: 2.94; *n* = 48; Ctrl: $\bar x$: 2.61; *n* = 32; *P*-value = 0.0009. Arrowheads point to cell nuclei in select fibres. ***P < 0.001, ns = *p* > 0.05. Error bars in **d**, **h**, **k**, **o**: SEM. Scale bars: 50 μm. Source data are provided (see ‘Data availability’).

**Fig. 5:**
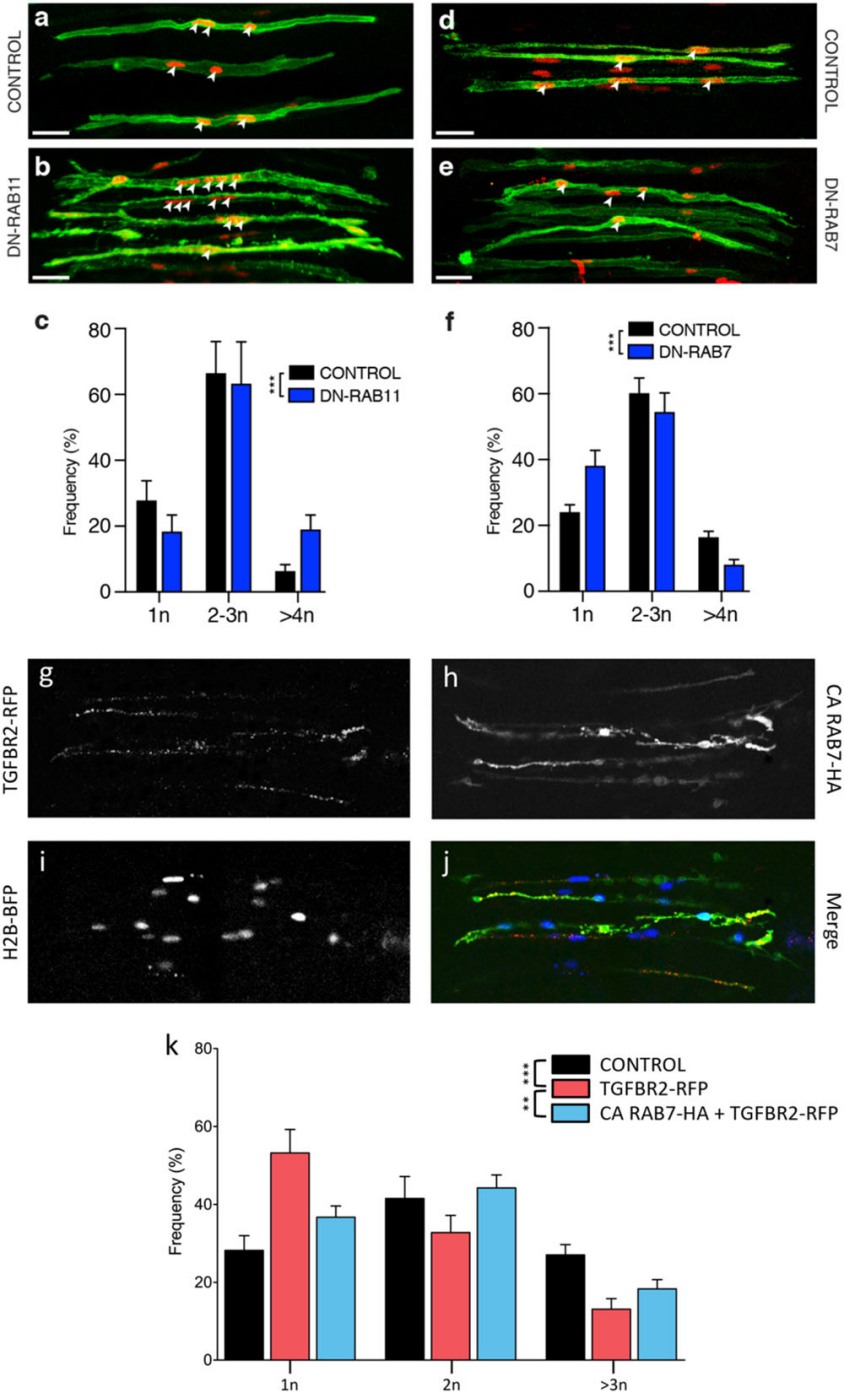
RAB and TGFβ signalling cooperate to regulate fusion. **a**, **b**, **d**, **e** DML-derived myocytes electroporated with MLC promoter driving expression of dominant negative (DN) variants of RAB11 (**b**) and RAB7 (**e**), together with membrane GFP (green) and nuclear mCherry (red). **c**, **f**, Column graph for **a**, **b** and **d**, **e** showing the population of electroporated myocytes containing the indicated number of nuclei relative to their controls (in %). **g**–**i** Functional rescue experiment where DML-derived myocytes were co-electroporated with a RFP-tagged form of TGFBR2 and an inducible (Tet-on) HA-tagged form of a constitutively active RAB7. **g** immunostaining against RFP showing the punctated expression of TGFBR2. **h** immunostaining against HA showing the diffuse expression of CA RAB7 (after Doxycyclin treatment). **i** Native fluorescence of H2B-BFP fusion protein, showing the nuclei within electroporated myocytes. **j** Merge of Fig. 5g–i. **k** Column graph showing the population of electroporated myocytes containing the indicated number of nuclei relative to their controls (in %) in each of the indicated conditions. Statistical analyses: DN RAB11: $\bar x$: 2.57; *n* = 14; Ctrl: $\bar x$: 2.07; *n* = 15; *P*-value <0.0001; DN RAB7: $\bar x$: 1.96; *n* = 19; Ctrl: $\bar x$: 2.46; *n* = 35; *P*-value < 0.0001; TGFBR2: $\bar x$: 1.63; *n* = 15; Ctrl: $\bar x$: 2.05; *n* = 23; *P*-value <0.0001; TGFBR2 + CA RAB7: $\bar x$: 1.84; *n* = 27; *P*-value=0.0019. ***P < 0.001. **P < 0.01. Error bars in **c**, **f**, **k**: SEM. Scale bars: 50 μm. Source data are provided (see ‘Data availability’).

To address the role of the pathway in myoblast fusion, we then performed a series of experiments using the MLC promoter driving the expression of various constructs, co-electroporated with membranal EGFP and a nuclear mCherry to evaluate the number of nuclei per electroporated myofibre^9^. Statistical analyses were performed for each experiment on the populations of counted myofibres (mean and *P*-values indicated in the text below). Graphic representations of those data (scatter plots with mean and standard deviation) are presented as Supplementary Figs. (6–12 and 14–17). To better illustrate our results, we show in Figs. 3–5 the same data, but distributed into three classes (i) fibres containing one nucleus; (ii) fibres with 2–3 nuclei; (iii) fibres containing 4 or more nuclei. A higher proportion of fibres containing one nucleus indicates a tendency to arrest fusion, while a higher proportion of fibres containing 4 or more nuclei indicates a tendency to promote fusion. Solid colours in column graphs indicate significant differences with controls while striped patterns indicate non-significant results.

Overexpression of SMAD4 by electroporation in the DML resulted in a significant (***P-value; Supplementary Fig. 6) decrease in fusion in the population of DML-derived myocytes 3 days after electroporation (E5.5), at a time-point where fusion is largely under way throughout the myotome (Fig. 3a, b, e). In fact, a majority of electroporated myocytes remained mononucleated (+84%, compared to controls), while we observed a large deficit of fibres containing more than 4 nuclei (−57%, compared to controls). Similarly, overexpression of SMAD3 resulted in a significant decrease (***P-value; Supplementary Fig. 6) in fusion when compared to controls (Fig. 3c, e). As with SMAD4, many SMAD3-expressing fibres remained mononucleated (+134%, compared to controls), while only few multinucleated fibres were observed (−96%, compared to controls). SMAD7 is a pan-inhibitor of TGFβ signalling (R-SMAD2/3- as well as R-SMAD1/5/8-dependent)^27,28^. Electroporation of SMAD7 in DML-derived myocytes led to a remarkable over-fusion phenotype (***P-value; Fig. 3d, e; Supplementary Fig. 6). Nearly all fibres had 4 or more nuclei (+512%, compared to controls) and only few were mononucleated (−91%, compared to controls). Even though BMP (R-SMAD1/5/8-dependent) signalling did not modify C2C12 fusion, we ruled out their implication in myoblast fusion in vivo by electroporating constitutively active SMAD1 and SMAD5 in DML cells. Three days later, the nuclei count of electroporated myofibres was not significantly different from controls (Supplementary Fig. 7; Fig. 3f–i). This confirms that BMP signalling does not play a significant role in the fusion of myoblasts to myofibres during early myogenesis. Similarly, even though MSTN did not act on C2C12 fusion, we verified that the same holds true in vivo. We have electroporated the MSTN inhibitor FST^16^ in the DML. Three days later, the nuclei count of electroporated myofibres was not significantly different from controls (Supplementary Fig. 8), thereby ruling out a function of MSTN in myoblast fusion. While the experiments described above indicate that TGFβ (R-SMAD2/3-dependent) signalling inhibits fusion in vivo, it was important to confirm these data by loss-of-function approaches. We used the CRISPR-mediated gene-editing technique we recently applied to chicken embryos^29,30^ to target SMAD3 by electroporating the DML with a CRISPR/Cas9 vector containing two gRNAs directed against the chicken SMAD3 sequences. Results showed that loss-of-function of SMAD3 within muscle fibres significantly increased fusion (***P-value; Supplementary Fig. 9), compared to a CRISPR Cas9 vector containing no gRNAs (Fig. 3j, k, m), resulting in a decrease (−30%, compared to controls) in mononucleated fibres and an increase (+29%, compared to controls) in multinucleated myocytes. Since SMAD2 and SMAD3 can independently mediate TGFβ receptor transcriptional activity^31^, we then targeted both molecules using the CRISPR technology. We co-electroporated the DML with two CRISPR/Cas9 vectors containing gRNAs directed against SMAD3 and SMAD2 sequences. Results showed that their simultaneous loss-of-function within muscle fibres improved the results obtained with only the CRISPR SMAD3 (***P-value; Supplementary Fig. 9), resulting in a decrease (−43%, compared to controls) in mononucleated fibres and an increase (+57%, compared to controls) in multinucleated myocytes (Fig. 3l, m). This suggests that SMAD2 and SMAD3 cooperate to regulate the transcriptional inhibitory activity of TGFβ receptors on myoblast fusion. Altogether, these results demonstrate that, in somites, SMAD2/3-dependent TGFβ signalling acts as a molecular brake on the fusion machinery. Inhibition of the pathway unleashes its restraint, resulting in a boost in the rate of DML-derived myofibres fusion, while its constitutive overexpression increases its constraint, leading to a near complete arrest of fusion.

### A receptor complementation mechanism mediates TGFβ activity

The cellular mechanism(s) whereby TGFβ signalling may regulate fusion in the embryo is suggested by the expression patterns of TGFβ family members in early somites (Fig. 1f–i) and the known fusion behaviour of different populations of cells. During early myogenesis DML-derived myofibres fuse only to cells emanating from the anterior and posterior borders of the dermomyotome^9^ (Supplementary Fig. 3). Thus, the fusion of (TGFBR2-positive) progenitors emanating from the anterior and posterior borders of the dermomyotome, onto (TGFB2/3- and TGFBR1-positive) myocytes derived from the DML, should, through a receptor complementation process, bring all TGFβ pathway components together, thereby allowing the activation of its signalling, and resulting in a decrease/arrest of fusion.

To test this appealing hypothesis, we first evaluated whether TGFBR1 is regulating the fusion of DML-derived myocytes to progenitors originating from the anterior and posterior borders of the dermomyotome. The expression of MLC-driven constitutively active (CA) and dominant negative (DN) forms of TGFBR1 in DML-derived myocytes resulted in an overall significant reduction or increase, respectively, in their fusion when compared to controls (***P-value; Supplementary Fig. 10; Fig. 4a–d). We observed for CA TGFBR1 an increase (+22%, compared to controls) in mononucleated fibres and a decrease (−72%, compared to controls) in myocytes containing 4 and more nuclei (Fig. 4b, d). In contrast, DN TGFBR1 resulted in a decrease (−34%, compared to controls) in mononucleated myocytes and an increase (+95%, compared to controls) in large fibres (Fig. 4c, d). Similar to DN-TGFBR1, a CRISPR/Cas9-mediated loss-of-function against TGFBR1 significantly increased fusion (***P-value; Supplementary Fig. 11; Fig. 4f, h), resulting in a decrease (−49%, compared to controls) in mononucleated fibres and an increase (+98%, compared to controls) in large fibres. These experiments demonstrate that TGFBR1 is the type I receptor regulating fusion in somites. TGFBR1 heterodimerizes with TGFBR2 to transmit signals from TGFβ ligands (TGFB1,2,3; Myostatin and GDF11), intracellularly^10^. If TGFBR2 expressed by anterior and posterior border-derived cells were responsible for triggering the TGFβ pathway and inhibiting fusion in DML-derived fibres, its loss-of-function in anterior or posterior border-derived cells should lead to an increase of fusion, while its loss-of-function in DML-derived cells should have no effect. Similarly to the DML cell population, the anterior or posterior borders can be specifically targeted by electroporation^23^. We first electroporated the DML with a CRISPR/Cas9 construct against TGFBR2; quantification of fusion showed that this did not significantly modify fusion when compared to controls (Supplementary Fig. 11; Fig. 4g, h). In contrast, electroporation of the same CRISPR/Cas9 TGFBR2 construct in the posterior border (PB) showed a significant increase in overall fusion of myocytes (***P-value) with a decrease (−13%, compared to controls) in mononucleated fibres and an increase (+46%, compared to controls) in large myocytes containing 4 or more nuclei (Supplementary Fig. 12; Fig. 4i–k). These data support the hypothesis that in trunk somites, the regulation of fusion by TGFβ signalling relies on the merging of TGFBR2-positive posterior border cells to TGFBR1-positive DML-derived cells.

If this were true, TGFβ signalling would be triggered only when fusion of these two cell populations is taking place. A first attempt to monitor the activity of the pathway during fusion using published SMAD3 reporters (based on SMAD-binding elements^32^) was unsuccessful, as this tool was inefficient at detecting TGFβ activity in somites. It was shown that SMAD binding to DNA is significantly enhanced by cell-type-specific master genes, such as Oct4 in ES cells or MYOD in muscles^33^. We tested the ability of genomic DNA sequences co-occupied by SMAD3 and MYOD^33^ to serve as TGFβ reporter during muscle fusion. We identified a (280 bp) sequence containing (6) MYOD- and (2) SMAD3-binding consensus sequences that faithfully detected TGFβ activity in a myogenic context (Supplementary Fig. 13a–i). Coherent with the expression data of TGFBR2 and TGFBR1 described above, we observed that the reporter was virtually inactive (0.5% electroporated cells were positive for the reporter) in progenitors present in the posterior border of the dermomyotome (Fig. 4l, o). One day later (at E3.5), as posterior border cells have translocated into the myotome and are aligned along the myocytes emanating from the DML, but have not yet fused to them^9^, the activity of the reporter was present in a minute proportion (4.5%) of the electroporated cells (Supplementary Fig. 14; Fig. 4m, o). In sharp contrast, the vast majority (89%) of electroporated cells expressed the reporter two days later (at E5.5), when vigorous fusion is taking place (*9;* Fig. 4n, o). These data demonstrate that the activation of TGFβ signalling is triggered by the fusion of posterior border-derived cells to DML-derived myofibres.

Based on the above, we propose a model whereby the fusion of anterior or posterior border myoblasts and their fusion to DML-derived myocytes results in the activation of the TGFβ pathway by a unique mechanism of receptor heterodimer complementation. As the activation of the pathway leads to an inhibition of fusion, the merging of a border myoblast to a DML-derived myofibre is likely to trigger a fusion-refractory state in the receiving myofibre.

### Fusion competence is restored by RAB

Since all myofibres within the myotome eventually become polynucleated, we reasoned that the inhibition of fusion described above should only be temporary. For additional rounds of myoblast fusion to occur, it is crucial that TGFβ signalling decreases within the receiving myofibre. Thus, we asked whether myoblast fusion-refractory and fusion-competent states could be controlled by cycling TGFβ receptor activity. Like other cell surface receptors, TGFβ receptors are rapidly internalized after activation. The main mechanism of TGFBR internalization is via clathrin-dependent endocytosis^34^. The small GTPase RAB proteins are key regulators of intracellular membrane trafficking of growth factor receptors^35^. Both type I and type II TGFβ receptors are recycled back to the cell surface by recycling endosomes, through a RAB11-dependent mechanism. On the contrary, lysosomal degradation, mediated by a RAB7-dependent mechanism, terminates signal transduction. Inhibiting RAB11 function should thus lower receptor recycling at the cell membrane, and ultimately decrease receptor signalling. Conversely, inhibiting RAB7 function should reduce receptor degradation, resulting in an extension of signal transduction. To test this, we electroporated a dominant-negative form of RAB11 (under the MLC promoter) in DML-derived fibres. Three days later, this resulted in a significant increase (***P-value) in fusion when compared to controls (Supplementary Fig. 15; Fig. 5a–c), resulting in a decrease (−34%, compared to controls) in mononucleated fibres and an increase (+203%, compared to controls) in large fibres. In contrast, electroporation of DN-RAB7 in DML-derived fibres led to a significant reduction (***P-value) in fusion when compared to controls (Supplementary Fig. 16; Fig. 5d–f), resulting in more (+59%, compared to controls) mononucleated fibres and less (−51%, compared to controls) in large myocytes. Altogether these results suggest that in muscle fibres, the recycling or conversely, the degradation of TGFβ receptors play important roles in the regulation of myoblast fusion to myofibres.

It was possible that RAB molecules modulate fusion by interacting with other molecular or cellular mechanisms than the ones suggested by our study. To support the hypothesis that RAB and TGFBR cooperate to regulate fusion, we performed a functional rescue experiment. We electroporated a WT form of TGFBR2 (driven by CAGGS ubiquitous promoter) in the DML of E2.5 embryos, together with an inducible (Tet-on), constitutively active form of RAB7 (CA RAB7). Given the reported role of RAB7 in TGF receptor degradation, demonstrating that the effect of overexpression of TGFBR2 on fusion is compensated by the overactivation of RAB7 would be a strong indication that the two molecules cooperate in this process. In the absence of doxycyclin, only TGFBR2 was over-expressed (Fig. 5g, j). Similarly to the experiments described above where SMAD3, SMAD4 or CA TGFBR1 were electroporated (Figs. 3b, c and  4b), this activated the pathway, resulting in a significant inhibition of fusion (***P-value; Supplementary Fig. 17). This was observed as a considerable increase in mononucleated fibres (+89%) and a decrease in large fibres (−52%), compared to controls (Fig. 5k). The addition of doxycyclin to developing embryos activated the expression of CA RAB7 (Fig. 5h, j). This resulted in a partial rescue of the inhibition of fusion observed with TGFBR2 alone (Supplementary Fig. 17) with a decrease in the number of mononucleated fibres (−31%) and an increase in large fibres (+40%), compared to TGFBR2 only (Fig. 5k). These experiments show that triggering RAB7 reverses the inhibition of fusion resulting from the overexpression of TGFBR2. This does not rule out the possibility that RAB7 may act in a number of molecular contexts, including with other receptors or other regulators of fusion, but it provides evidence that RAB7 can cooperate with TGFBR signalling to regulate myoblast fusion in early myogenesis. Altogether, these data support the hypothesis that consecutive fusion-refractory and fusion-competent states are controlled by TGFβ signalling, effectively regulating the pace of myoblast fusion in vertebrate embryos.

## Discussion

**Fig. 6:**
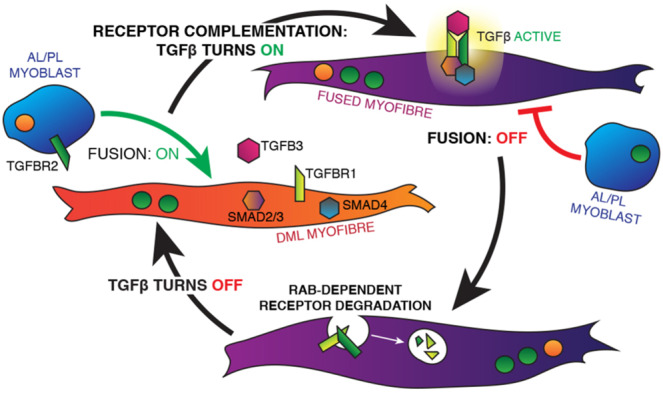
A model describing the receptor complementation mechanism. Prior to fusion, myocytes originating from the DML (in orange) express the ligand TGFB3, the effectors SMAD 3, & 4 and the receptor TGFBR1. They are competent to fuse. Myoblasts originating from the posterior or anterior borders of the somite (in blue) express TGFBR2. Upon fusion of myoblasts to DML-derived myocytes, membrane merging of the fusion partners allow the activation of the TGFβ pathway by a receptor heterodimer complementation mechanism (in purple), ultimately leading to a temporary inhibition of fusion of another myoblast. The process of fusion is likely reinitiated after RAB-mediated receptor degradation.

This study demonstrates (Fig. 6) that TGFβ signalling plays a unique role in myoblast fusion during early embryogenesis. In contrast to the previously identified genes belonging to the machinery necessary for fusion, the TGFβ pathway acts as a molecular brake in this process. We had previously observed that during early embryonic development, the rate of muscle fusion in trunk was about 2.5 times slower than in limb. It is likely that TGFβ signalling, alone or in combination with other molecules, participates in the differential rate of fusion in these two body regions. Significant differences in nuclei numbers are also observed within individual developing muscles (e.g. the limbs), where fibres containing the highest number of nuclei are preferentially localized in the centre of the muscle masses while shortest are found in the periphery^9^. Differential fusion may thus have direct consequences on the patterning of muscles during development and it is likely that this process is regulated by a variety of signals and pathways. The hyper-fusion phenotype that we obtained when inhibiting TGFβ signalling and the observation that many tested genes inhibited the fusion of C2C12 in vitro suggests that tight harnessing of fusion at multiple molecular checkpoints is an unsuspectedly crucial aspect of muscle formation. It is also likely that aberrant fusion may impact muscle function. While the chicken embryo is not appropriate to test the functional consequence of deregulated TGFβ signalling, a contemporaneous study from F. Le Grand’s laboratory^36^ suggests that TGFβ-mediated deficit in fusion results in functional impairement of mouse regenerating muscles, while over-fusion does not improve their function.

Our observation that, in vivo, TGFβ signalling does not act on early myogenic differentiation contrasts with a wealth of data published decades ago on the strong inhibitory effect of TGFβ signalling on the myogenic differentiation of primary myoblats and cell lines in vitro (e.g. refs. ^18–20,37^), and that we confirmed in the esiRNA screen on C2C12 presented above. Whether the in vivo*/*in vitro discrepancy is due in vitro conditions (culture conditions, substrate rigidity) that poorly represent the in vivo environment^38–40^ or to specific characteristics of the muscle progenitor population targeted in this study is only speculative at this point. However, the demonstration that the TGFβ pathway plays an identical function in adult mice^36^ supports the hypothesis that the inhibitory function of TGFβ signalling on myoblast fusion is critical to many if not all aspects of muscle patterning, growth, function and repair in vertebrates.

The in vivo electroporation in the chicken embryo allows an exquisite precision in targeting specific cell populations within somites. The mechanism (that we named receptor complementation) is unique as it relies on the fusion of progenitors to myofibres, which allows receptor dimer complementation and signalling to occur. The resulting inhibition of fusion suggests that auto-inhibition may be the mechanism whereby fusion controls itself. The reversible nature of receptor signalling (through RAB-mediated receptor degradation) completes this mechanism, allowing it to reset. Therefore, membrane fusion, signalling and receptor degradation likely collaborate in a reiterating regulatory module that ultimately controls whether fusion takes place and at what pace.

Altogether these findings uncover that muscle progenitors, progressing through the myogenic program and thereby acquiring fusion competency, are confronted with an additional level of regulation, epitomized by TGFβ signalling. Acting as a molecular brake, it expands the possibilities of temporal and spatial control of muscle patterning, from a simple stop or go signal to the fine-tuning of fusion that likely governs crucial phases of muscle growth and regeneration. This opens new routes of investigation into an unforeseen aspect of muscle biology that could bring important benefits to muscle therapies.

## Methods

### EsiRNA screen generation and candidate selection

We performed a genome-wide RNA interference (RNAi) functional screen on a mouse C2C12 muscle cell line (Fig. 1). The strategy was based on the transfection of long dsRNA enzymatically digested by bacterial RNase III for the generation of esiRNA. This process generates a heterogeneous population of siRNAs capable of interacting with multiple sites on the target mRNA: since each single siRNA in a pool has different off-targets while having the same on-target, the use of siRNA pools dilutes out off-target effects^41–43^. The esiRNA library comprised 8627 mouse genes. An EGFP-positive C2C12 cell line was transfected with esiRNAs, and they were placed in differentiation medium. Three days later, the detection of fused myotubes was performed, using an automated cell-recognition imaging program (Definiens) that quantified the number of nuclei (stained with Hoechst) per fibre (detected by the EGFP green fluorescence). Since a lack of C2C12 fusion upon esiRNA transfection could be a true effect on cell fusion or due to a failure to differentiate, the wells were also stained for myogenin to evaluate the myogenic differentiation at the end of the experiment. The total number of nuclei per well also gave an indication that the esiRNAs did not interfere with cell proliferation or survival. In each plate, a number of control wells served to normalize the results obtained in each assay. They were transfected with esiRNA against Renilla and Firefly Luciferase (RLuc and FLuc, negative controls) and against the Rac Family Small GTPase 1 and the Cell Division Cycle 42 genes (RAC1 and CDC42, positive controls). We have performed two rounds of transfection and each esiRNA was tested in triplicate. The readings were normalized (to 100) to the effect of negative control esiRNAs (RLuc and FLuc) assayed on each multi-well plate. As expected, transfection of RAC1 and CDC42 esiRNAs led to a significant decrease in the fusion of C2C12 (RAC1: −27%; CDC42: −48% compared to controls, Table 3). The esiRNA library, the transfection, the immunostainings and image analyses were performed by Cenix Bioscience in close collaboration with our group. All subsequent steps, normalization, statistics and bioinformatics analyses were done in our team. Full data availability upon request.

### In situ hybridization and sectioning

In situ hybridizations were performed as described in ref. ^24^. RNA probes were generated from ESTs obtained from the chick EST database at BBSRC^44^. The following EST was used for TGFBR2: chEST327g19. Images for in situ hybridizations of TGFB2, TGFB3 and TGFBR1 were obtained with permission from the GEISHA database^45^. Embryos were either analysed after whole-mount staining or sectioned into 15 µm slices using a Leica cryostat (as described in ref. ^46^).

### Chicken electroporation

Fertilized eggs were obtained from a local farm. No ethical approval is needed for work on chicken young embryos. The somite electroporation technique that was used throughout this study has been described previously^25,47^. In short, chicken embryos at stage 15–16 of Hamburger Hamilton (HH;^21^ 24–28 somites) were electroporated in the dorso-medial border (DML) or the posterior border (PL) of newly formed interlimb somites. The electroporation technique results in the mosaic expression (50% at best^9^) of the DNA constructs in the somite sub-domains (DML and PL). This is particularly important in the case of a study on muscle cell fusion, since the observed phenotypes result from the competition of electroporated and wild-type myoblasts for the fusion to recipient myofibres. This diluting effect is even more pronounced in the case of PL electroporation, since PL-derived myoblasts compete for the fusion to DML-derived myocytes not only with WT myocytes from the PL, but also with WT myoblasts from the AL. When a dominant-negative molecule (such as SMAD7) is over-expressed, this mosaicism should impact less, if at all, upon the phenotype. This likely explains the important differences observed between the phenotypes obtained after CRISPR-mediated loss-of-function and after overexpression of SMAD7.

### Expression constructs

The CAGGS-transposase and the Tol2 MLC-nls-mCherry were described before^9^.

The following plasmids were created by inserting all sequences described below into a Tol2 MLC-IRES GFPcaax (i.e. an EGFP addressed to the plasma membrane by a CAAX-box prenylation domain). The mouse SMAD3, SMAD4, SMAD6 and SMAD7 were obtained from the I.M.A.G.E. consortium (distributed by Source BioScience). A mouse SMAD5 was obtained from pCMV5-Smad5 (gift from Jeff Wrana, Addgene plasmid #11744^48^). A constitutively active form (CA SMAD5) was generated by replacing Serine 463 and 465 by Aspartic Acid (S463D, S465D). A constitutively active form of the human SMAD1 (S463E, S465E) was obtained from pCS2 hSmad1-EVE (gift from Edward De Robertis, Addgene plasmid #22993^49^). A constitutively active form of the rat TGFBR1 (T202D) was obtained from pRK5 TGF beta type I receptor (gift from Rik Derynck, Addgene plasmid #14833^50^). A dominant-negative form of the human TGFBR1 (K232R) was obtained from pCMV5B-TGFbeta receptor I K232R (gift from Jeff Wrana, Addgene plasmid #11763^51^). A dominant negative form of the human TGFBR2 (K277R) was obtained from plasmid pCMV5B-TGFbeta receptor II (a gift from Jeff Wrana and Joan Massagué, Addgene plasmid # 11762^52^). A WT human TGFBR2 was obtained from plasmid pCMV5B-TGFbeta receptor II WT (same origin as K277R^52^, Addgene plasmid #11766). It was RFP tagged in the stop codon. A dominant negative form of the human Rab11A (S25N) was obtained from plasmid PCMV-intron myc Rab11 S25N (a gift from Terry Hébert, Addgene plasmid #46786^53^). A dominant negative form of the human Rab7A (T22N) was obtained from plasmid DsRed-rab7 DN (a gift from Richard Pagano, Addgene plasmid #12662^54^). A constitutively active form of human RAB7 (Q67L) was obtained from plasmid EGFP-Rab7A Q67L (a gift from Qing Zhong, Addgene plasmid # 28049^55^). It was HA tagged in the stop codon and cloned into an inducible (Tet-on) bidirectional pBI vector^47^. All vectors are available upon request.

### CRISPR/Cas9 constructs

The use of CRISPR/Cas9 technology on somatic cells in chicken was based on refs. ^29,30^. Guide RNA design was done using the site CHOPCHOP: http://chopchop.cbu.uib.no/ (Cornell, Montague^56^). The gRNA sequences were as follow: for the chicken SMAD3: gRNA1 cSMAD3: gTCATCTACTGCCGGCTGTGG (targets exon 2); gRNA2 cSMAD3: gTCCACTCGTTGGTAATGATA (targets exon 2); for the chicken TGFBR1: gRNA1 cTGFBR1: caccGTAACTCCATCTCTGAAGGA (targets exon 2); gRNA2 cTGFBRR1: caccgAGTATTGGTAAGGGTCGCTT (targets exon 4); for the chicken TGFBR2: gRNA1 cTGFBR2: caccGCTTCCTCTTCTTGTGAGTG (targets exon 4); gRNA2 cTGFBR2: caccGTGGATGACTTGGCCAACAG (targets exon 4). Two sites per gene were chosen (hence the two gRNA sites) in order to excise a portion of the coding sequence and promote the loss of function of the targeted proteins in electroporated cells. The expected genomic DNA deletions sizes are: cSMAD3: 136 bp; cTGFBR1: 8096 bp; cTGFBR2: 681 bp. The efficiency of the chosen gRNAs was tested by transfection of the constructs in DF1 chicken fibroblast cells in vitro (as described in refs. ^29,30^).

### Immunohistochemistry and imaging

Whole-mount antibody staining were performed as described in ref. ^24^. The following antibodies were used: rabbit polyclonals directed against RFP (Abcam #62341 and Sigma # AV34143, 1/1000), chicken polyclonal antibody against EGFP (Abcam #13970, 1/1000), mouse monoclonal directed against Myosin Heavy Chain MYH1 (MF20) (deposited to the DSHB by Fischman, D.A.), mouse monoclonal antibody directed against Follistatin (Santa Cruz sc-365003) and mouse monoclonal antibody directed against HA (Abcam #1424). Embryos were washed and cleared in 80% glycerol/PBS. Whole-mount embryos were imaged using a Leica SP5 confocal microscope with scanning resonance running LAS AF software (Leica MicroSystems). Image stacks were analysed by using either the Imaris software package (Bitplane, version 7.5.2) or ImageJ software.

### ChIP-seq data analyses for Smad3 and MyoD in myotubes

ChIP-seq data for SMAD3, MYOD and IgG Control were mapped to mouse reference genome (mm10) using Bowtie2 (version 2.1.0﻿;^57^) with the following settings: *bowtie2 -k2 -N1 -L32–end-to-end -p 4 --phred33 -x Bowtie2Index -U ChIP.fq 1*>*ChIP.sam 2*>*ChIP.log* We selected one replicate for SMAD3 and one replicate for MYOD based on sequence quality scores and percent read alignment. Peaks were called by using MACS2^58^ with the following setting: *macs2 callpeak -t sample.bam -c Igg_control.bam -f BAM -g mm -n sample --bw 300 -q 0.01 -B*. The SMAD3 regions with the 20 highest peaks were selected. Of these peaks, we chose the 10 that overlapped with highest peaks for MYOD.

### Quantification and statistics

Manual counting of nuclei per myocytes within confocal z-stacks was performed using the cell counter plugin of ImageJ^59^. Statistical analyses were performed using the GraphPad Prism software. Each condition comprised about 6 embryos from two independent experiments. An average of 22 distinct somites per condition (17 total) were evaluated (somite number = n in the text). The number of counted myocytes per experimental condition was about 525 fibres (total of 8925 counted fibres). Mann–Whitney two-tailed non-parametric tests were applied on the entire population of counted myofibres to evaluate the significance of each treatment (P-values are indicated in the text). Experimental treatments (gain or loss of gene function) that led to significant differences compared to controls are indicated on graphs by solid colour columns; non-significant results are indicated by striped colour columns; controls are indicated by black columns. The column graphs in Figs. 1–4 depict the population of electroporated myocytes that were calculated for each experimental condition. They were distributed in three groups, myocytes that contained one nucleus, those that contained 2–3 nuclei and those containing 4 or more nuclei, expressed as a percentage of the entire myocyte population. Tendencies to decrease or promote fusion are more pronounced in the first and last (1N and >4N) categories. Therefore, we describe in the text the changes, compared to controls, in the proportion of fibres in these two categories. Columns are accompanied by the standard error of the mean (SEM). ***P-value < 0.001 extremely significant; **P-value 0.001–0.01 very significant; *P*-value > 0.05 non-significant.

### Reporting summary

Further information on research design is available in the Nature Research Reporting Summary linked to this article.

## Supplementary information

Supplementary Information

Reporting Summary

## Data Availability

The source data for all figures are available at https://figshare.com/articles/dataset/Source_Data_Melendez_Excel_xlsx/13240970 for the raw data and at https://figshare.com/articles/dataset/Source_Data_Melendez_Prism_pzfx/13241135 for the statistical analyses. Links to publicly available datasets: Chick EST database at BBSRC: http://www.chick.manchester.ac.uk/; GEISHA chicken embryo gene expression database: http://geisha.arizona.edu/. ChIP seq data supporting the findings of this study have been deposited in the National Center for Biotechnology Information Gene Expression Omnibus (GEO) and are accessible through the GEO Series accession number GSE21614.
