# Supplementary material for: TGFβ signalling acts as a molecular brake of myoblast fusion
Source: Nat Commun. 2021 Feb 2;12:749. doi: 10.1038/s41467-020-20290-1 (PMC7854724; doi:10.1038/s41467-020-20290-1)
Supplement: Supplementary file 1 — Supplementary Information [file 41467_2020_20290_MOESM1_ESM.pdf]

TGFβ signalling acts as a molecular brake of myoblast fusion

SUPPLEMENTARY INFORMATION

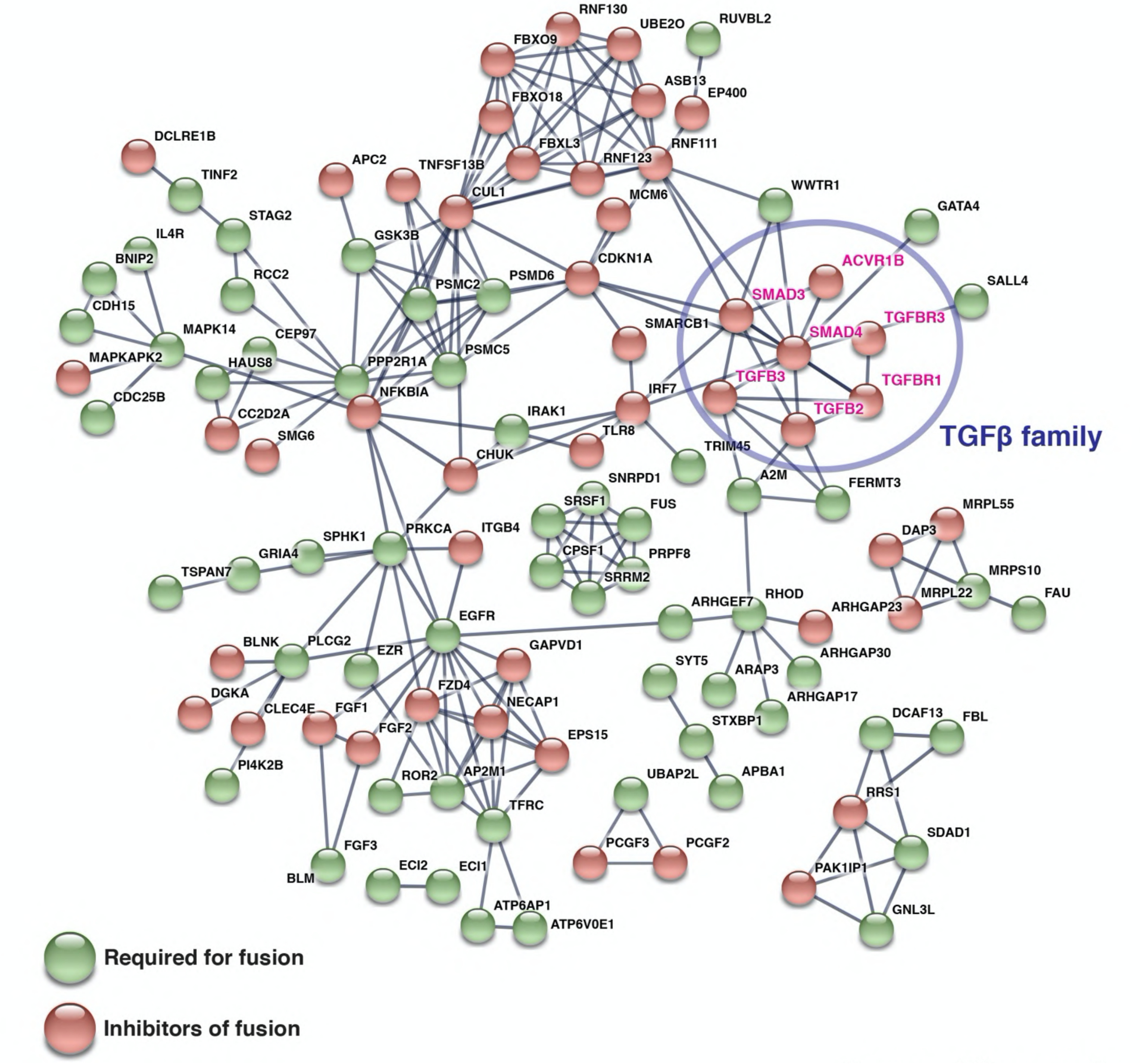

Supplementary Fig. 1

Representation of the predicted protein-protein interactions between candidate genes inhibitory (in red) and necessary (in green) for C2C12 myoblast fusion. The 200 most significant genes for each category were analysed through String software[40], using highest confidence interaction score. Disconnected nodes were hidden from final graph. Fusion-inhibitory and -necessary molecules were pseudo-coloured manually using Adobe Illustrator. Statistical overrepresentation tests (PANTHER and INGENUITY) on Gene Ontology molecular functions identified the TGFβ family as overrepresented (circled).

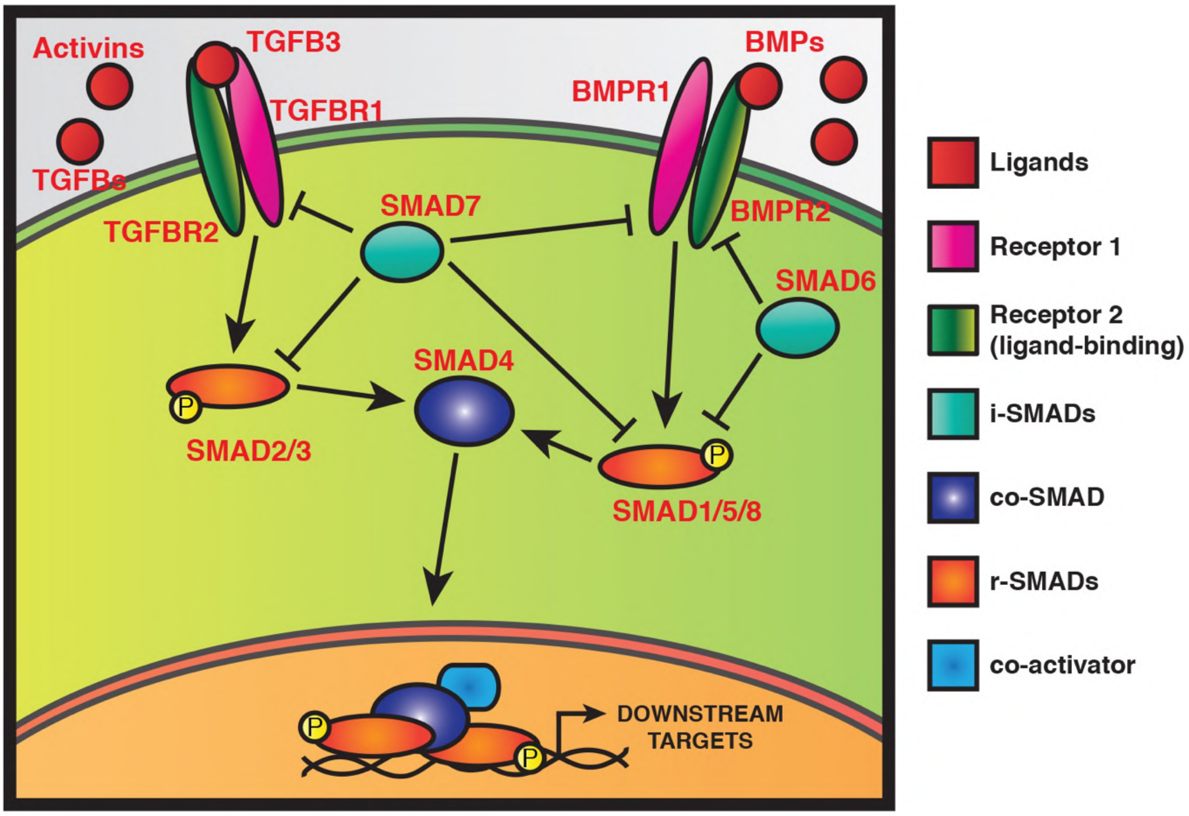

Supplementary Fig. 2
Diagram depicting essential molecules involved in canonical TGFβ signalling. TGFβ ligands (BMPs, GDFs, TGFβs, among others) bind type 2 receptors, which in turn recruit a type 1 receptor to form a hetero-tetrameric complex. Ligand-bound receptor 2 phosphorylates the serine residues of the receptor 1, which in turn phosphorylates the effector proteins R-SMADs (Receptor-regulated SMADs). BMP receptors only activate SMADs 1/5/8, while TGFBs, Activins and GDFs act through SMADs 2/3. Phosphorylated R-SMADs have a high affinity to SMAD4. The SMAD complex enters the nucleus and binds co-factors and transcription promoters to induce DNA transcription. I-SMADs (Inhibitory SMADs) are involved in negative feedback. SMAD7 competes for receptor 1 binding sites and prevents phosphorylation. SMAD6 competes with SMAD4 exclusively through the BMP SMAD1/5/8 interactions. Diagram has been simplified for clarity and relevance.

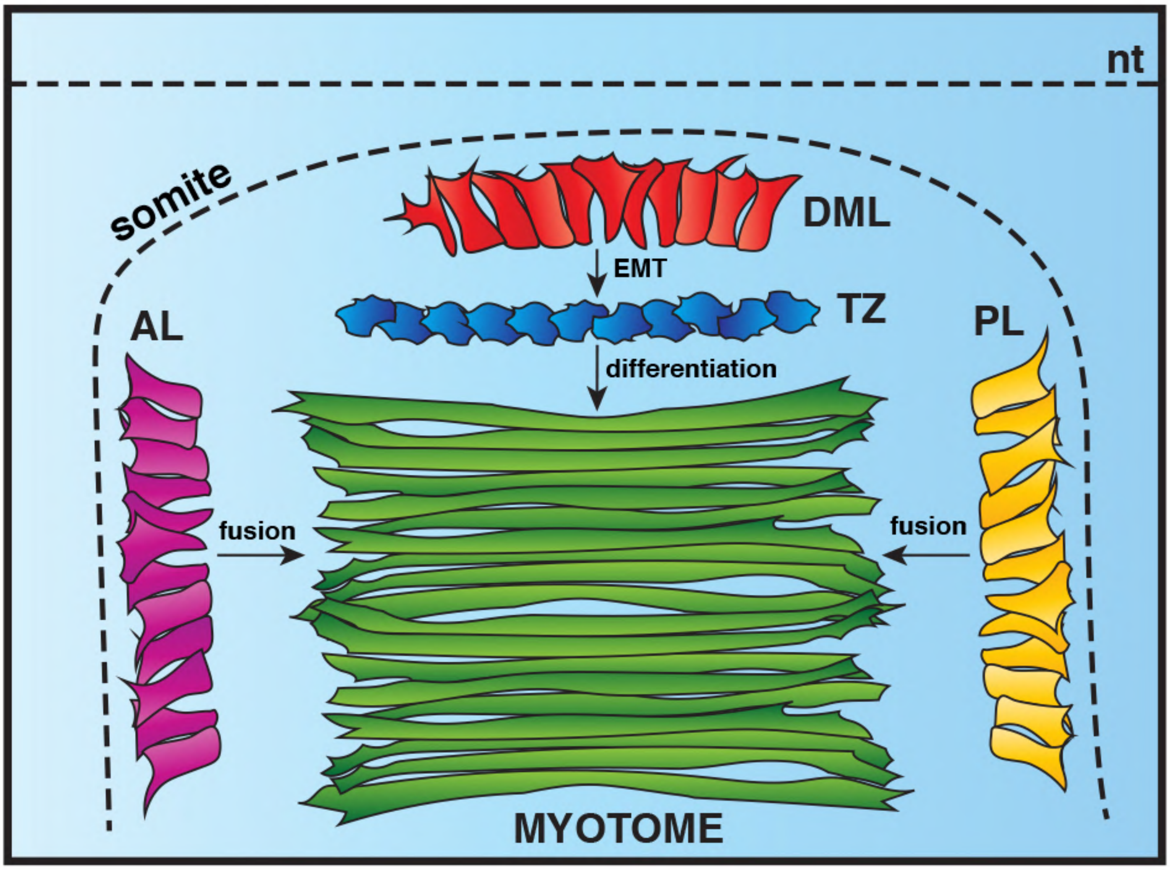

Supplementary Fig. 3
Diagram depicting the somitic regions taking part in the formation of the myotome and the fusion of myocytes. Selected epithelial cells originating from the medial border of the dermomyotome (DML) undergo an epithelial to mesenchyme transition (EMT) that allows their translocation in a region located beneath the dermomyotome, the transition zone (TZ), where they orient in the antero-posterior axis of the embryo[23–25]. The EMT triggers the entry of cells derived from the DML into the myogenic program. Terminal myogenic differentiation (e.g. MyHC expression) is observed when cells attach to the anterior and posterior borders of somites, at which time they are named myocytes (green elongated fibres). Fusion of myocytes is observed about 24 hours after myotome formation was initiated. Progenitors from the anterior (AL) and posterior (PL) border of the dermomyotome translocate in the myotome where they fuse to existing myocytes[9]. nt: neural tube.

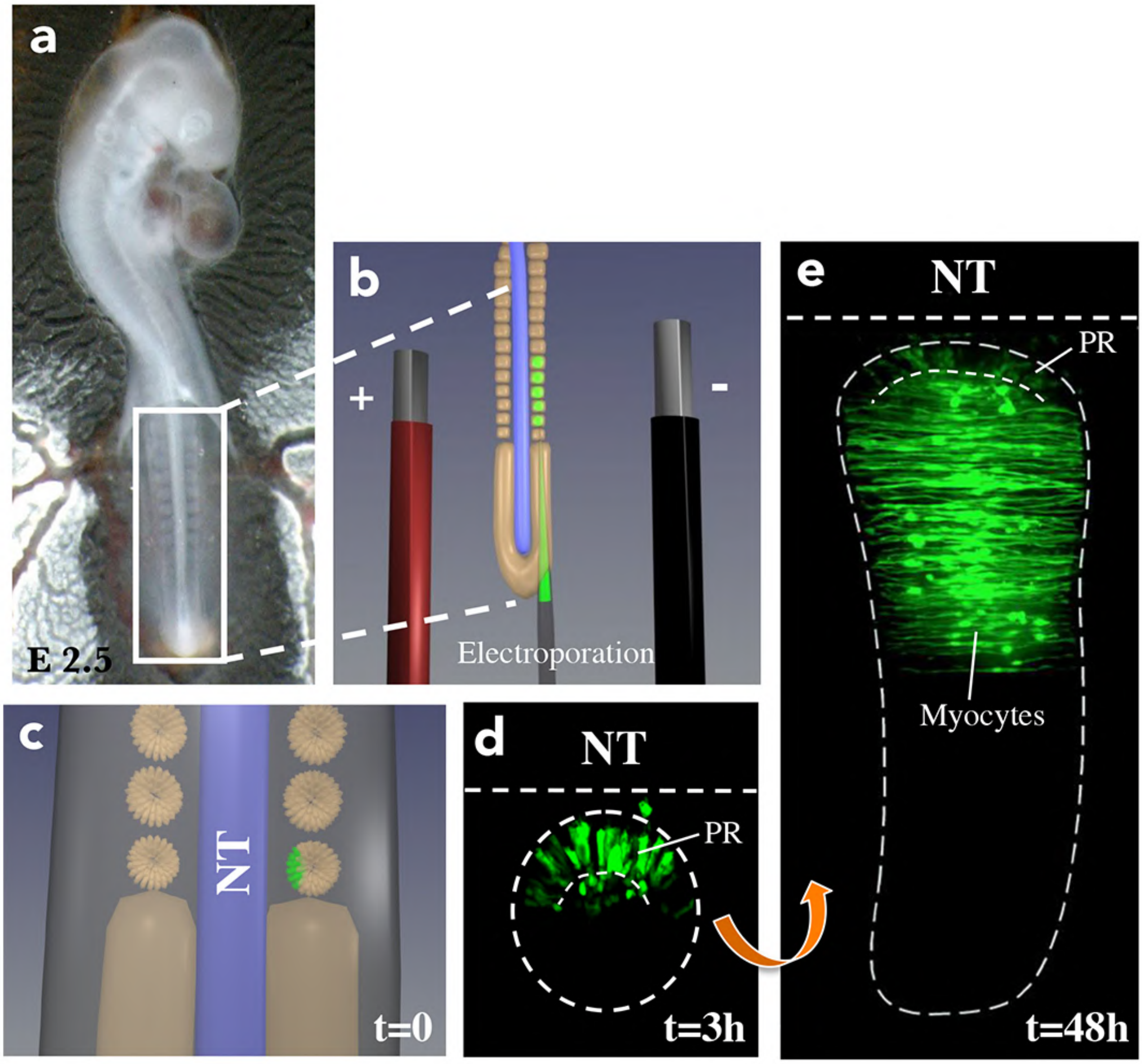

Supplementary Fig. 4
Electroporation protocol used throughout this study. Chicken embryos at 2.5 days of incubation (a) were electroporated in the medial border of newly formed somites (b,c). **d,** shows the epithelial progenitors (PR) expressing a GFP reporter driven by a CAGGS promoter in as short as 3 hours. **e,** Dorsal view of a somite, showing the myocytes that were generated during the two-day incubation time. NT, neural tube.

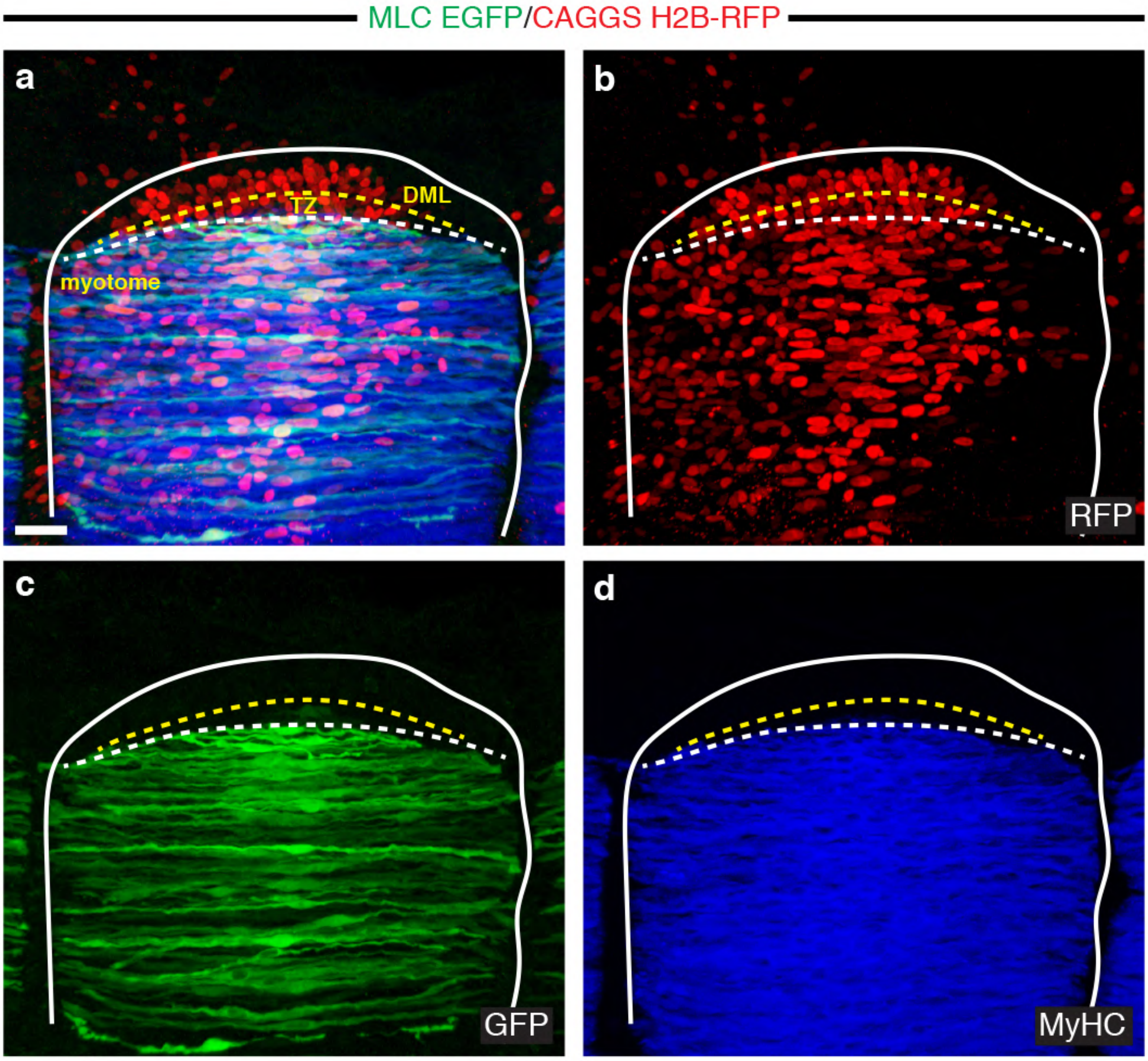

Supplementary Fig. 5

Myosin Light Chain promoter is expressed in terminally differentiated myocytes. a, Overlay confocal stacks of a single somite electroporated at HH15 (E2.5), and imaged at HH25 (E4.5). The white solid line delineates the somite. The dotted lines delineate the transition zone (TZ). **b**, Electroporation control, a CAGGS ubiquitous promoter driving nuclear H2B-RFP. Expression is observed in the dorsomedial lip (DML), the TZ and the myotome. **c**, Expression of GFP driven by a myosin light chain (MLC) promoter. No expression of the reporter is seen in the DML. Cells within the DML initiate MYF5 and MYOD expression[25] before entering the TZ. TZ cells are all postmitotic and express MyoG (our observation). Faint GFP expression was observed in a few cells of the TZ, while all myocytes of the myotome robustly expressed the fluorescent marker. MLC is therefore activated in terminally differentiating progenitors; **d**, Antibody staining against Myosin Heavy Chain (MyHC) showing expression exclusively in differentiated muscle fibres of the myotome. RFP and GFP were detected with IHC. Scale bars: 50μm.

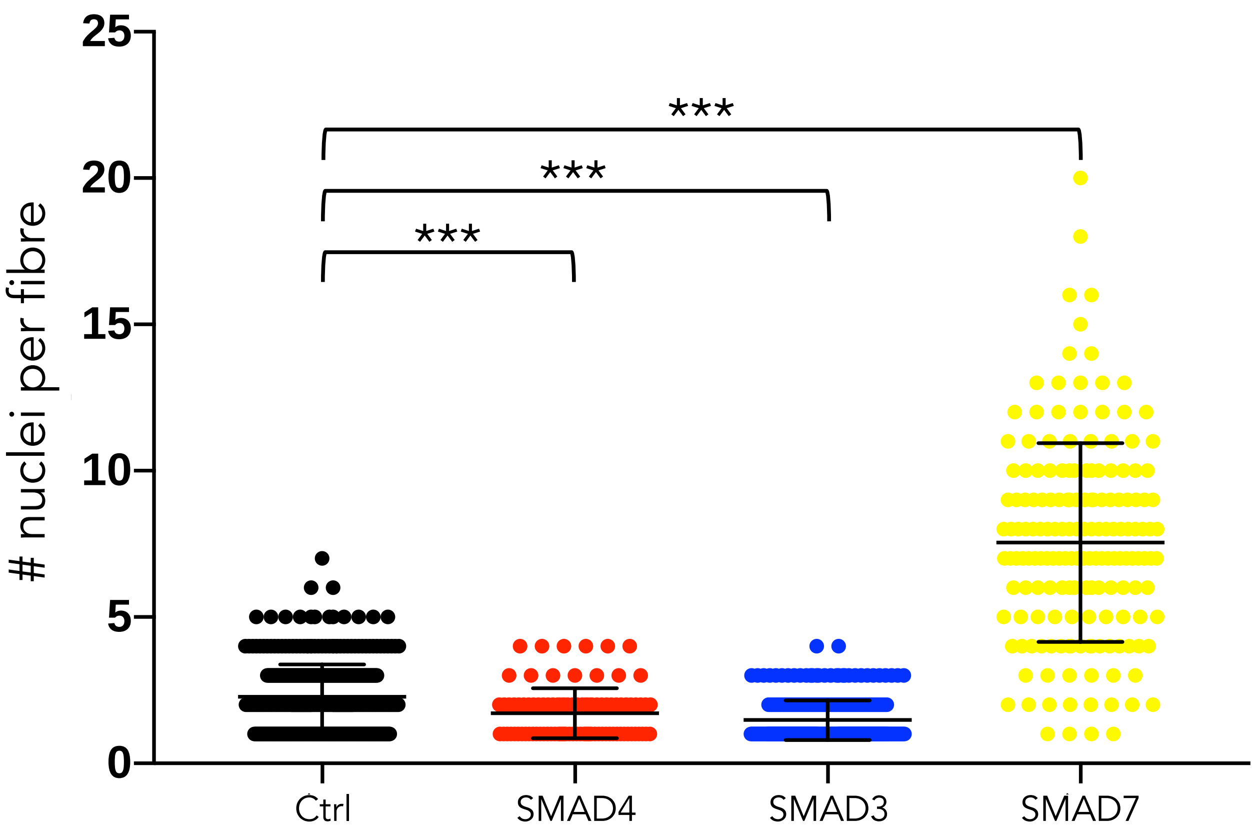

Supplementary Fig. 6
SMAD2/3-dependent TGFβ signalling regulates myoblast fusion *in vivo*. Scatter plots showing the number of nuclei per fibre in each condition. Shown are means and standard deviations; ***P<0.001

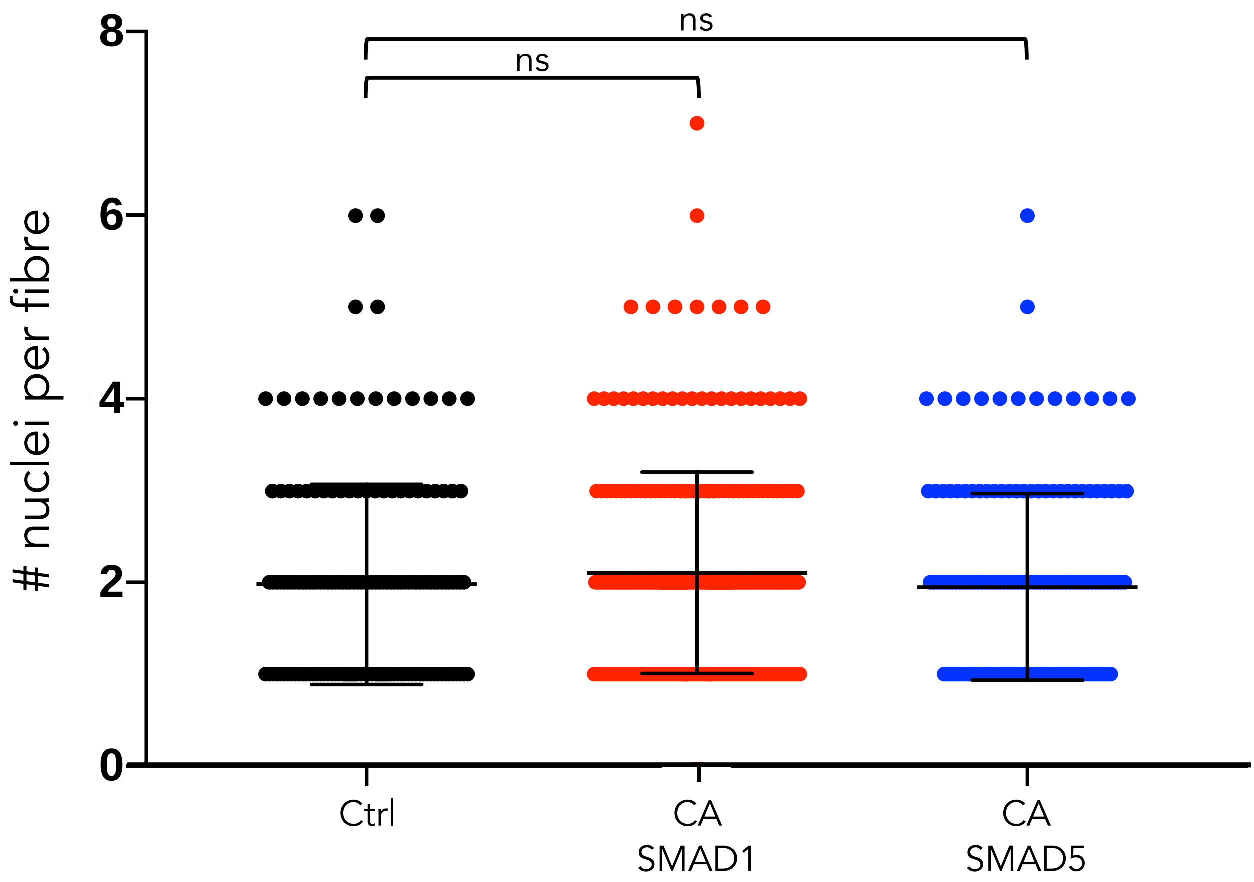

Supplementary Fig. 7

SMAD1/5/8-dependent TGFβ signalling does not regulates myoblast fusion *in vivo.* Scatter plots showing the number of nuclei per fibre in each condition. Shown are means and standard deviations; ns: non statistically significant difference.

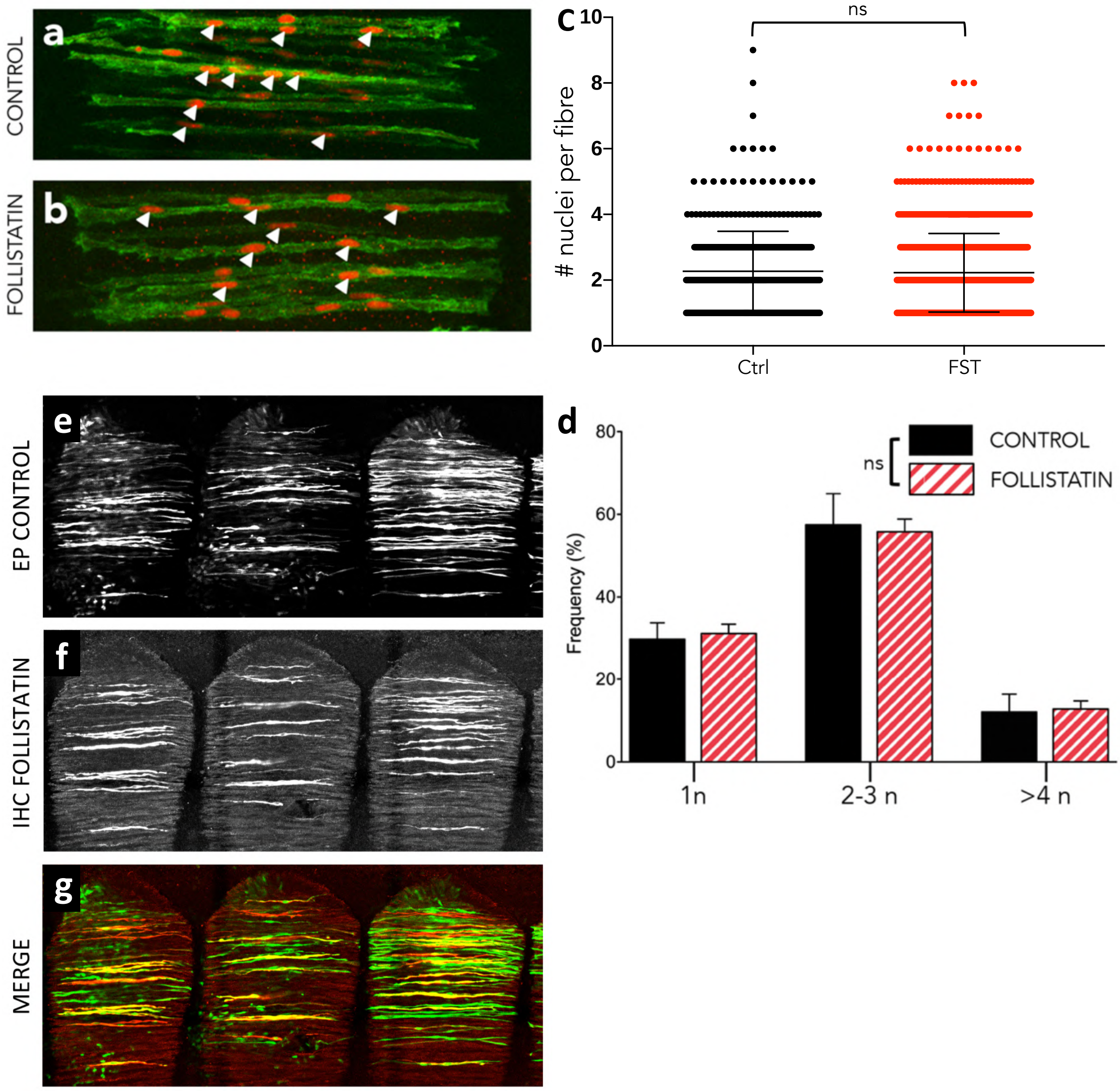

Supplementary Fig. 8

Myostatin does not regulate myoblast fusion *in vivo*. a,b, Dorsal views of confocal stacks of somites observed at E5.5 and electroporated in the DML at E2.5 with a plasmid coding for the Myostatin specific inhibitor Follistatin. Embryos were fixed and stained against GFP and RFP antibodies. **c,** Scatter plots showing the number of nuclei per fibre (means and standard deviations indicated). **d,** column graph for a,b showing the population of electroporated myocytes containing the indicated number of nuclei relative to their controls (in %). Error bars: SEM. White arrowheads point nuclei within fibres. **e-g,** Dorsal views of confocal stacks of somites observed at E4.5 and co-electroporated with a plasmid coding for BFP (as electroporation control) and for Follistatin and immunostained for FST (e). in **g,** merge. Statistical analyses: FST: $\overline{x}$: 2.23; n=53; Ctrl: $\overline{x}$: 2.27; n=20; P-value 0.46; Source data are provided (see Data Availability).

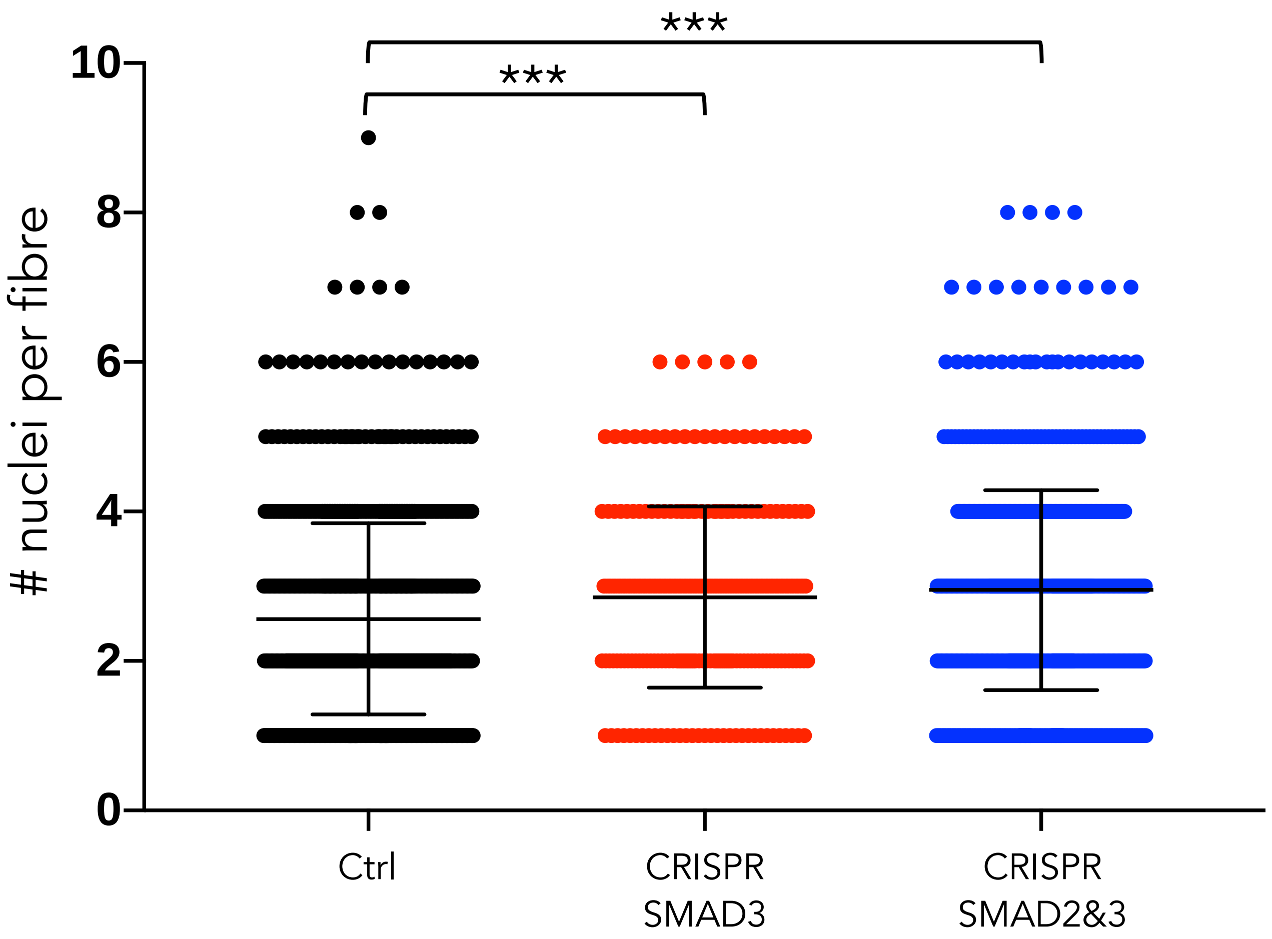

Supplementary Fig. 9
CRISPR-mediated loss of SMAD2 & 3 functions. Scatter plots showing the number of nuclei per fibre in each condition. Shown are means and standard deviations; ***P<0.001

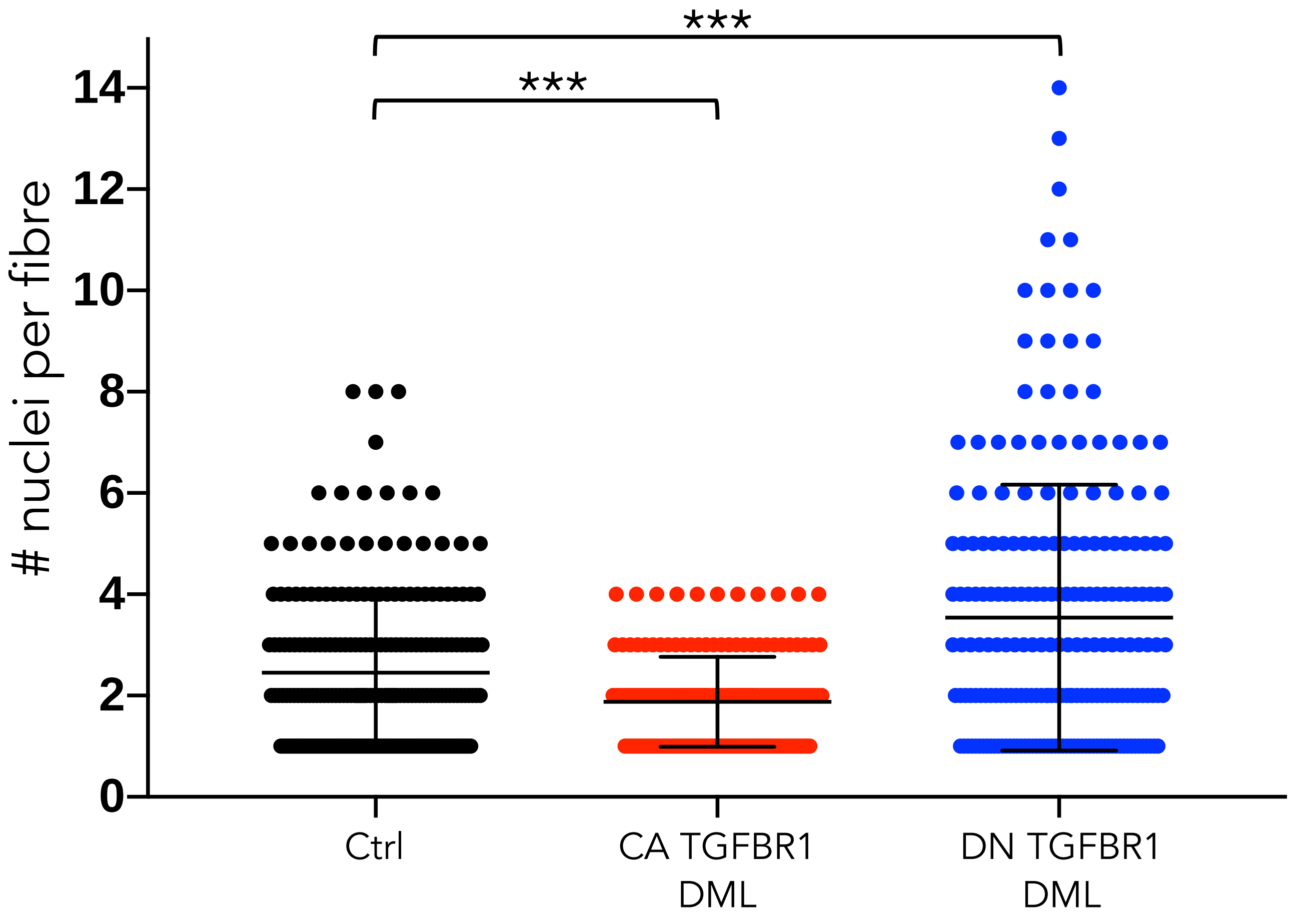

Supplementary Fig. 10
TGFBR1 in DML-derived myocytes regulates fusion. Scatter plots showing the number of nuclei per fibre in each condition. Shown are means and standard deviations; ***P<0.001

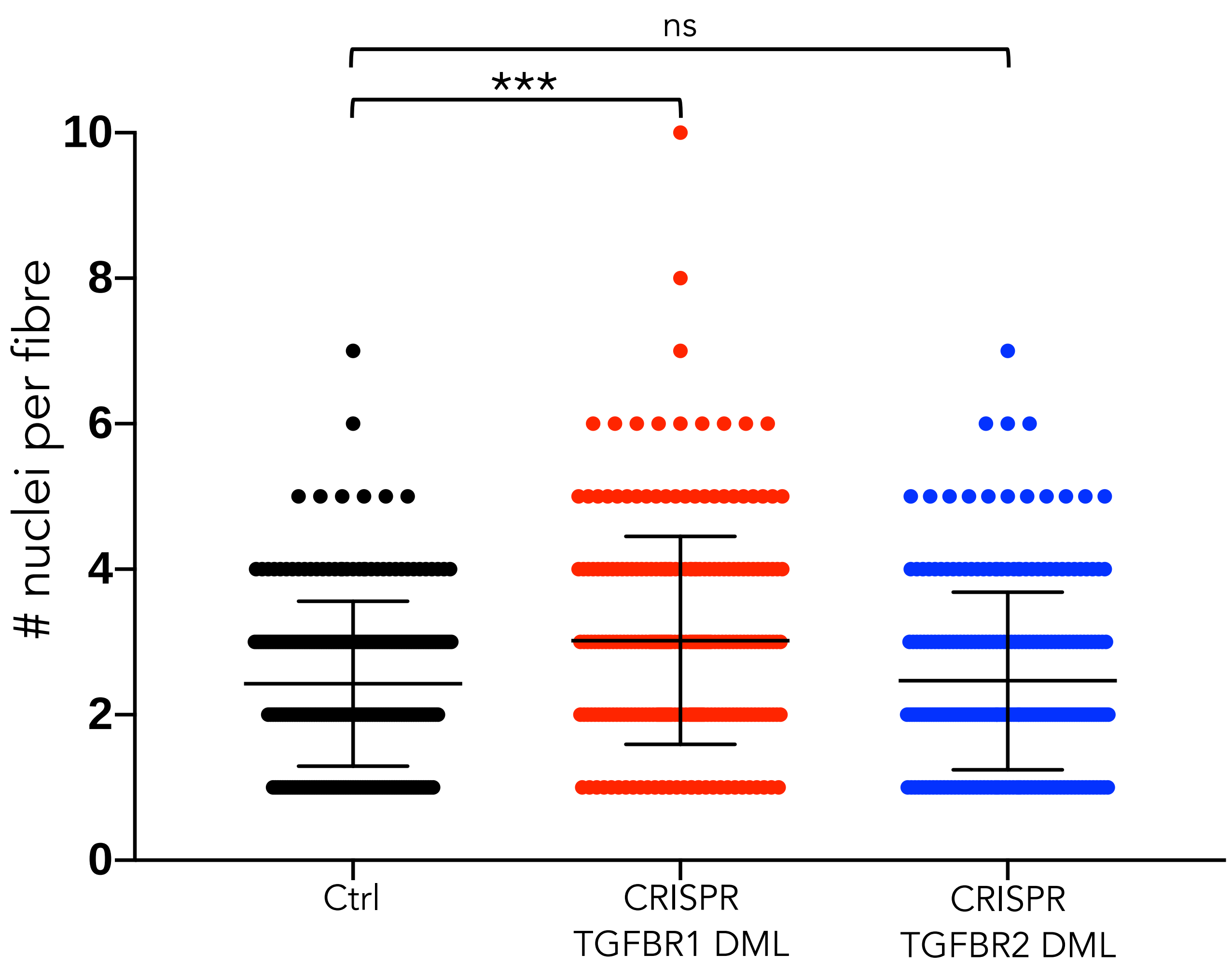

Supplementary Fig. 11

In the DML, TGFBR1 but not TGFBR2 regulates fusion. Scatter plots showing the number of nuclei per fibre in each condition. Shown are means and standard deviations; ***$P<0.001$; ns: non statistically significant difference.

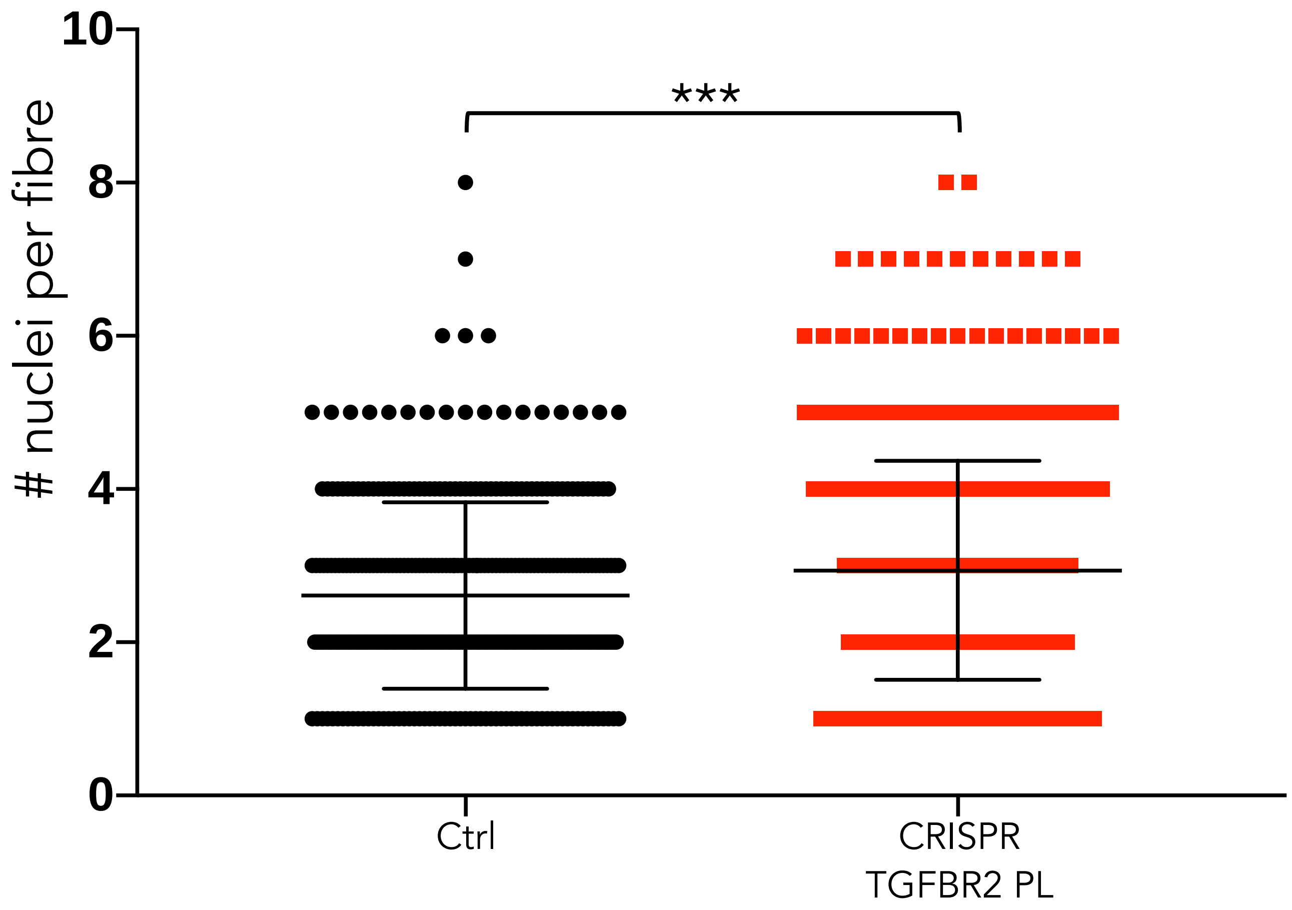

Supplementary Fig. 12
TGFBR2 in the posterior dermomyotome (PL) progenitors is necessary for fusion. Scatter plots showing the number of nuclei per fibre in each condition. Shown are means and standard deviations; ***$P<0.001$.

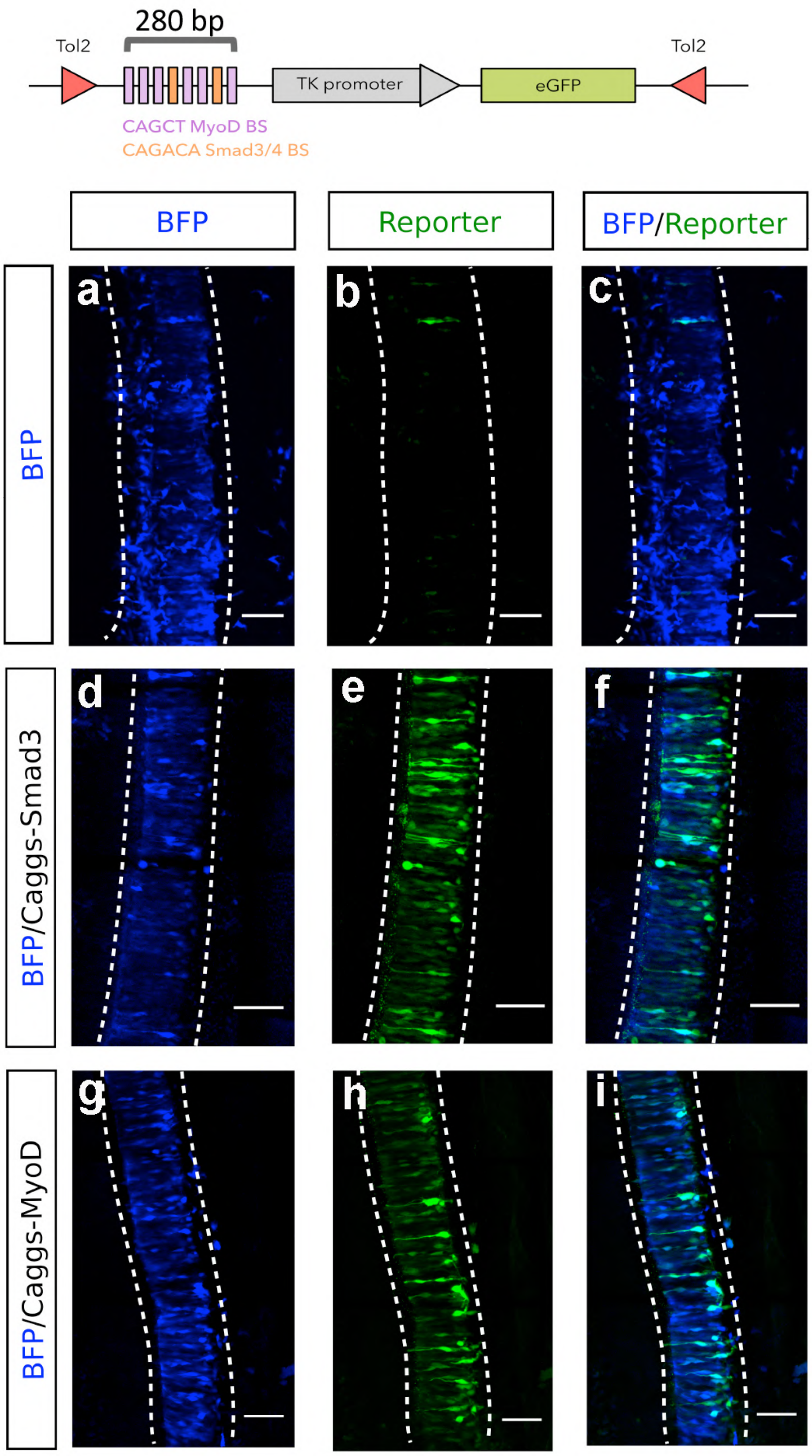

Supplementary Fig. 13

A new reporter to monitor SMAD3 activity in muscles. A 280 bp enhancer sequence located on mouse chromosome 11 between location 59161446 and 59161726, containing 6 MYOD and 2 SMAD3/4 binding sites, was cloned upstream of the TK minimal promoter and eGFP. **a-i,** Dorsal views of confocal stacks of one half of neural tube (delineated by dotted lines) co-electroporated with a plasmid coding for a CAGGS cytoplasmic BFP (in blue) and the reporter construct (in green). **d-f,** a CAGGS SMAD3 expression vector was added to the plasmid mix, resulting in a strong increase of the reporter activity. **g-i,** a CAGGS MYOD expression vector was added to the plasmid mix, also resulting in a strong increase of the reporter activity.

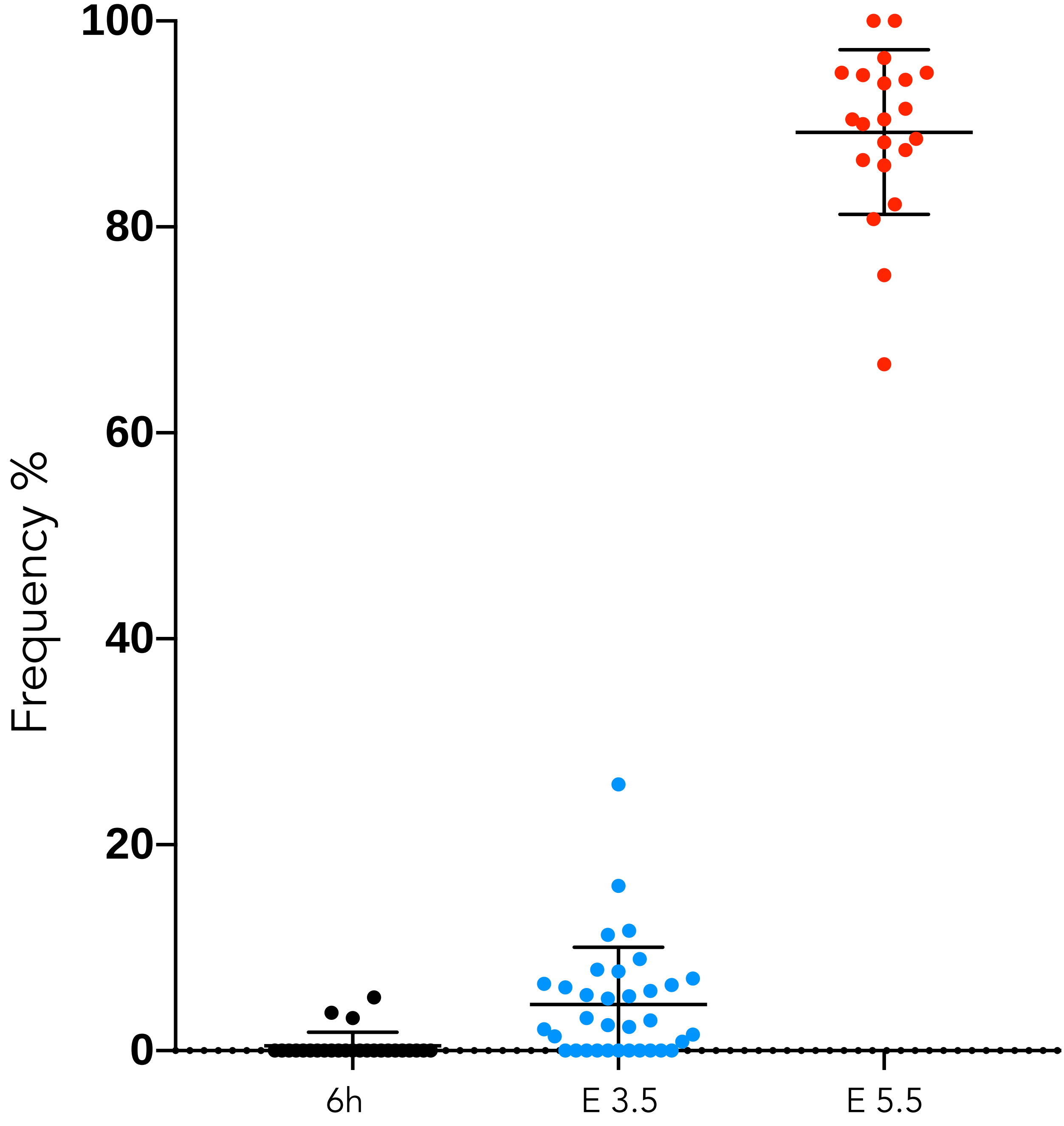

Supplementary Fig. 14

TGF signalling is activated upon fusion of PL-derived progenitors to DML-derived myofibres. Scatter plots showing the proportion of electroporated cells activating the TGF β reporter 6 hours, one day or 3 days after electroporation. Shown are means and standard deviations.

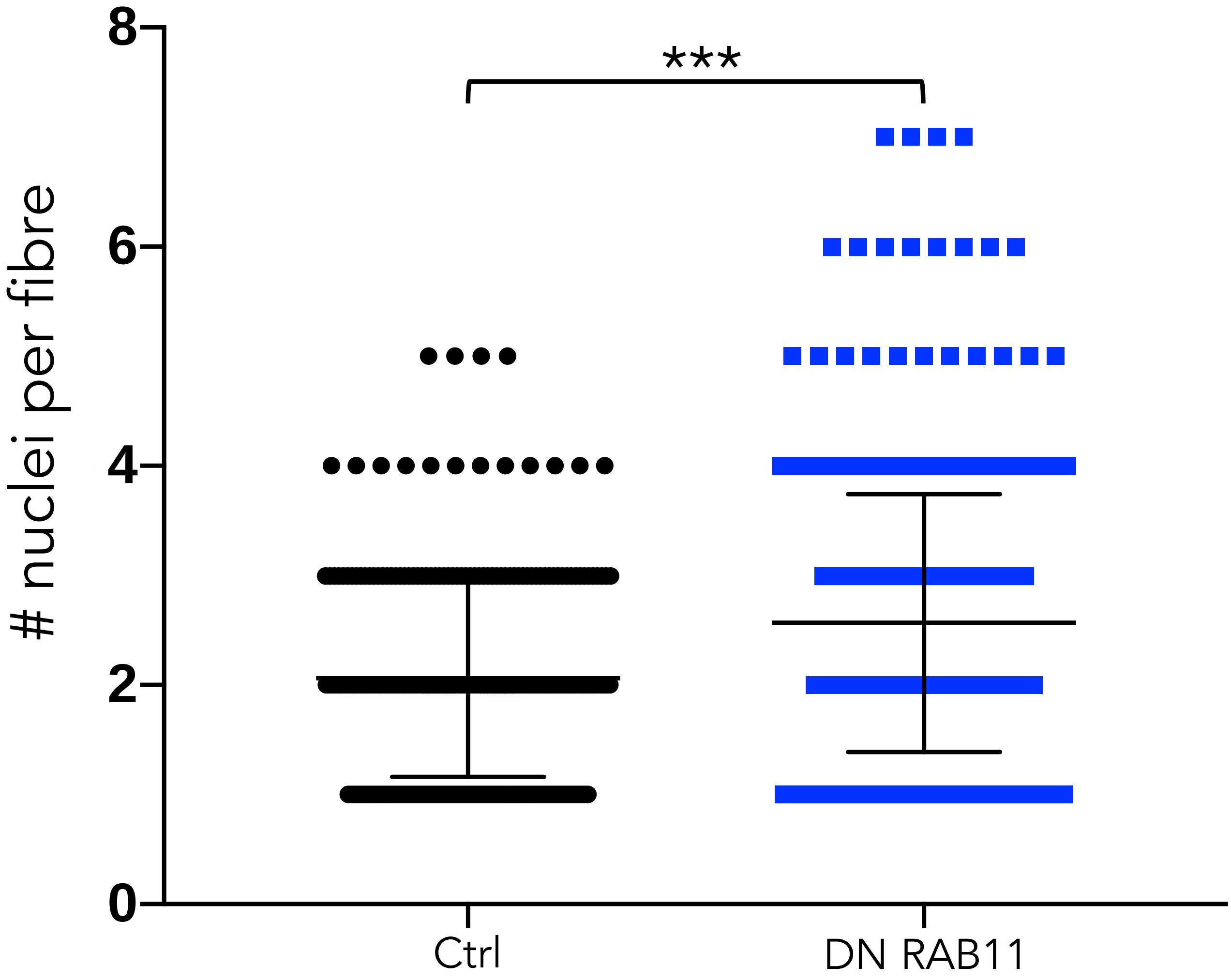

Supplementary Fig. 15

Myoblast fusion is dependent upon RAB11 function. Scatter plots showing the number of nuclei per fibre in each condition. Shown are means and standard deviations; $***P<0.001$.

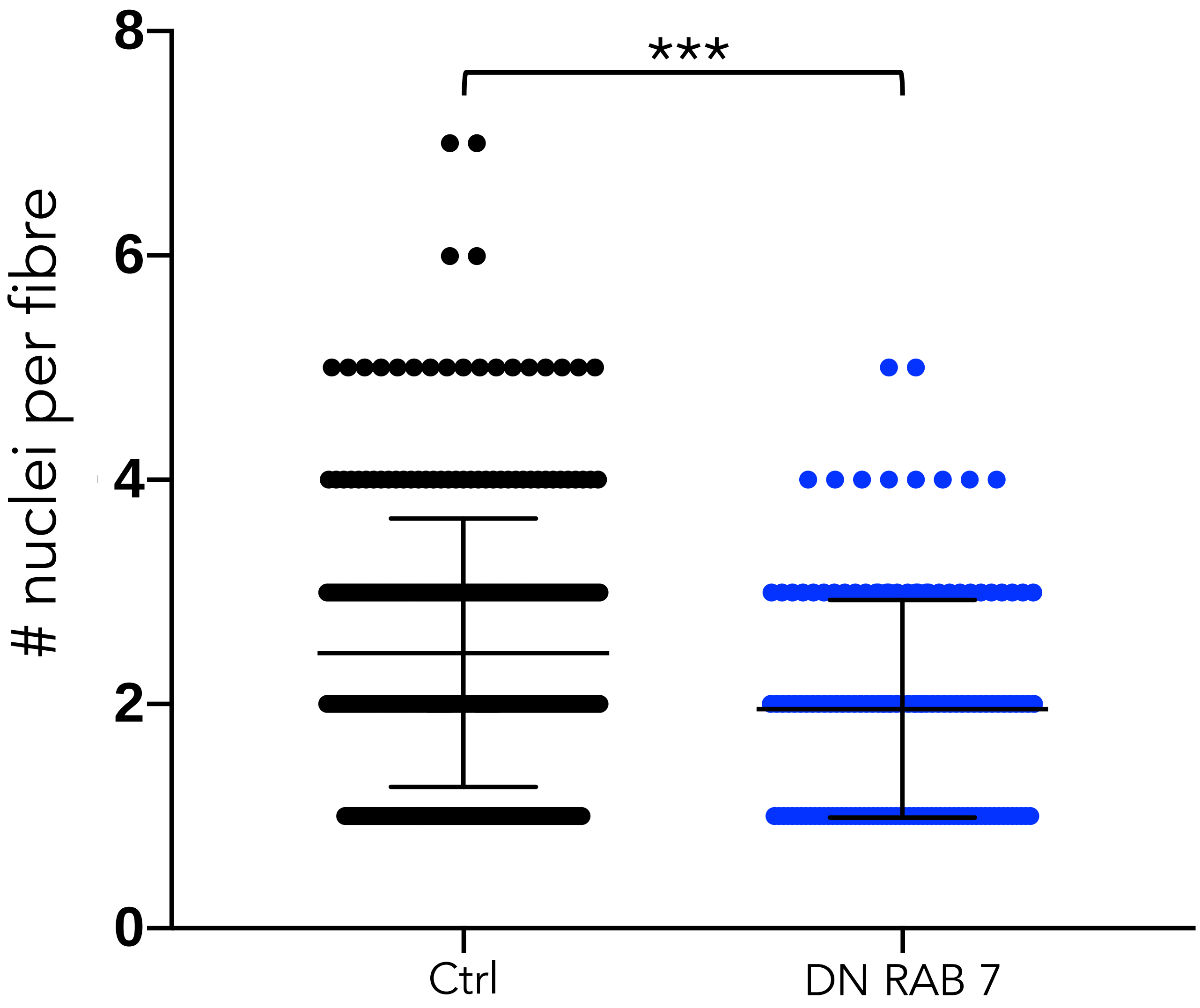

Supplementary Fig. 16
Myoblast fusion is dependent upon RAB7 function. Scatter plots showing the number of nuclei per fibre in each condition. Shown are means and standard deviations; ***P<0.001; **P<0.01.

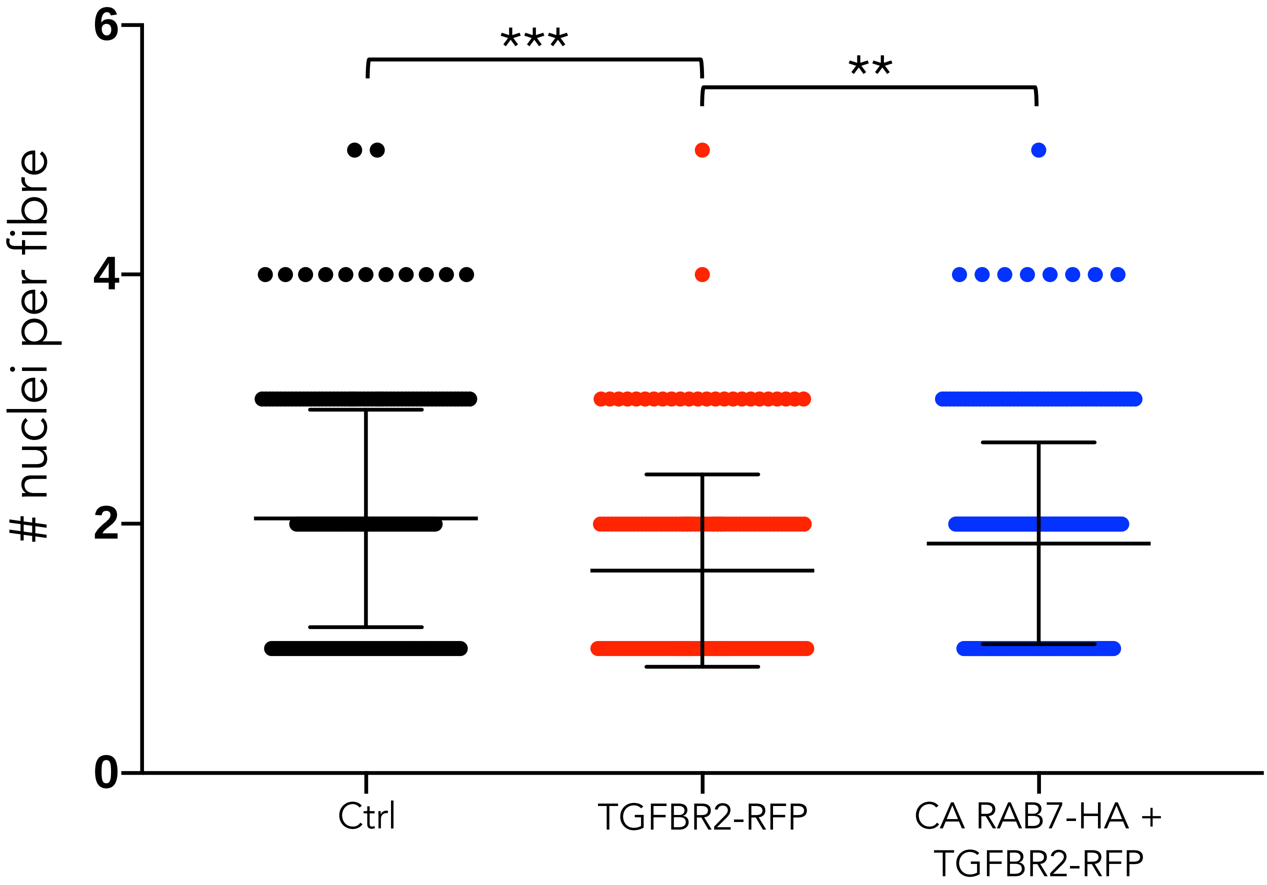

Supplementary Fig. 17

Rescue of TGFBR2-mediated inhibition of fusion by CA RAB7. Scatter plots showing the number of nuclei per fibre in each condition. Shown are means and standard deviations; ***P<0.001.

Table 1. Genes inhibitors of C2C12 fusion

Entrez Gene ID	pvalue	%nuclei/myotube	pvalue	%nuclei	pvalue	%myogenin
Smad4	8,42E-15	525,54	0,14	98,56	2,20E-16	156,57
Fzd4	2,20E-16	508,18	0,00	111,16	2,20E-16	139,43
Mdfi	2,20E-16	432,86	7,11E-15	148,72	6,41E-10	114,60
Tgfb2	6,13E-13	405,95	0,00	111,81	2,20E-16	154,19
Mmp17	2,52E-13	400,40	0,00	115,77	2,20E-16	145,97
Adh5	2,20E-16	387,35	1,21E-09	126,46	2,20E-16	125,79
Sgcd	3,77E-11	386,64	4,84E-07	119,80	0,01	106,67
Cyfip2	1,53E-05	382,66	0,00	92,34	0,00	104,30
2310002L13Rik	1,20E-15	379,80	2,79E-12	129,66	2,90E-09	115,36
Smad3	2,20E-16	377,95	0,73	100,32	2,20E-16	136,95
Habp4	1,84E-14	375,01	2,05E-07	85,37	2,20E-16	152,15
Mrpl55	1,66E-12	372,03	0,00	123,44	1,05E-13	132,64
Pard6b	2,20E-16	360,21	2,20E-16	145,19	0,08	104,93
Inpp4a	4,51E-09	348,96	1,96E-07	121,85	3,89E-13	130,06
D17Wsu104e	7,58E-13	348,92	2,80E-06	112,08	2,20E-16	138,39
Ube2o	9,79E-13	340,50	0,22	104,49	6,23E-08	83,31
Srp54a	8,88E-12	334,81	2,57E-12	119,38	1,74E-06	111,15
Spaca1	1,74E-14	329,89	5,73E-12	131,26	6,11E-16	136,73
Best1	2,20E-16	324,30	6,24E-05	110,93	0,08	93,83
Rnf111	8,86E-16	319,44	0,25	103,26	2,20E-16	136,57
3110062M04Rik	2,85E-14	307,52	1,87E-05	113,99	2,72E-16	130,69
Dnaic1	2,96E-15	304,79	1,16E-09	121,40	2,20E-16	135,97
Osbpl8	2,20E-16	300,28	6,92E-06	115,59	2,20E-16	143,16
Qpctl	1,96E-11	295,90	8,18E-09	127,91	3,18E-15	128,44
Capn7	2,20E-16	295,42	6,03E-09	116,37	2,21E-14	122,16
6330416G13Rik	7,27E-16	293,11	4,84E-10	117,67	2,80E-05	109,34
Tgfbr1	2,06E-13	284,01	0,56	97,57	2,20E-16	148,79
Rrs1	9,95E-12	280,82	4,41E-07	117,55	2,20E-16	145,62
Fis1	4,01E-12	280,12	1,90E-11	132,70	1,31E-13	72,58
Csrnp1	7,00E-13	276,67	3,40E-14	134,42	1,85E-11	121,61
Ep400	2,55E-13	276,09	0,71	99,15	4,31E-14	125,76
Mpv17l	1,33E-14	275,41	0,00	110,83	2,20E-16	148,62
2410018M08Rik	4,33E-15	274,92	0,01	107,24	2,20E-16	130,66
Pcgf2	6,21E-13	274,88	3,39E-11	119,28	2,20E-16	139,64
Tnfsf13b	1,76E-09	271,19	0,33	102,68	3,62E-16	125,97
Fam167b	2,31E-11	271,15	5,74E-13	130,48	7,14E-16	126,39
Scamp2	1,51E-13	269,26	3,09E-10	124,17	0,01	107,04
Wipi2	1,60E-09	268,12	0,00	115,89	2,20E-16	136,86
Slc9a4	1,30E-14	266,91	0,61	101,61	2,37E-11	118,18
Gja10	7,42E-15	266,80	1,15E-09	118,39	2,20E-16	130,90
Tgds	3,08E-15	266,63	6,14E-06	113,59	2,20E-16	151,78
Mixl1	3,32E-14	266,55	6,42E-10	120,73	2,20E-16	131,39
Ahsa1	3,65E-11	265,95	0,12	102,81	2,20E-16	138,02
Apc2	4,38E-15	263,90	1,64E-10	120,29	4,82E-16	126,99
Abca9	1,03E-12	262,27	6,74E-07	116,35	1,43E-15	127,14
Pcmtd2	6,76E-12	261,94	0,03	108,37	2,20E-16	133,32
Rab35	1,15E-13	258,54	2,20E-13	123,68	2,20E-16	141,86
Fam122b	4,42E-16	258,27	3,00E-04	109,98	2,20E-16	129,91
Smarcb1	3,34E-11	254,57	0,83	101,59	2,20E-16	140,38
Acvr1b	2,02E-15	254,50	8,15E-06	111,56	2,20E-16	124,43
Rundc3a	3,62E-12	253,83	6,91E-11	118,88	2,99E-11	116,02
Plekha3	2,20E-16	253,54	3,03E-06	113,11	4,13E-11	115,93
Necap1	2,00E-15	253,42	4,26E-13	122,07	2,20E-16	136,07
Olfr1189	1,17E-13	253,01	3,11E-06	114,19	2,20E-16	123,50
Ctps2	2,93E-06	252,82	0,00	115,25	2,20E-16	141,78
Lrit2	3,60E-16	252,38	0,00	112,53	3,84E-11	118,01
Gzf1	3,27E-13	252,11	7,47E-07	115,06	2,77E-15	124,70
Kcnj6	4,26E-12	249,45	1,85E-14	129,84	0,00	92,23
Asb13	6,09E-14	248,88	7,33E-07	113,51	8,29E-15	127,14
Nr1h5	1,94E-15	248,85	0,00	108,47	2,20E-16	135,59
Pcdhb7	3,28E-13	247,72	0,00	109,43	6,00E-16	129,21
Mcm6	3,02E-16	247,23	3,50E-09	118,13	2,20E-16	137,33
Alg5	2,02E-14	245,67	0,04	103,72	2,20E-16	138,85
Mut	7,92E-14	245,64	0,02	106,53	2,20E-16	141,59
Slc25a12	2,47E-13	245,47	4,34E-05	109,73	1,00E-15	125,34

Cbll1	6,92E-14	241,74	7,38E-05	111,77	2,43E-11	117,37
Ube4b	2,50E-12	240,76	0,36	97,13	0,13	103,00
Gdap2	1,92E-12	240,66	0,00	113,15	1,48E-12	121,31
Fgf1	8,63E-15	239,61	4,93E-16	128,92	2,20E-16	127,31
Rpap1	7,69E-15	238,91	7,64E-10	120,42	5,60E-08	112,73
Cul1	8,12E-11	236,19	0,02	105,28	2,14E-14	121,06
Ckap2l	1,42E-11	234,60	0,00	109,48	2,20E-16	142,69
Mrpl22	9,05E-15	234,45	4,00E-06	112,30	0,16	103,22
Bola3	1,60E-06	234,02	0,00	109,75	2,20E-16	138,64
Rufy3	1,11E-09	233,93	0,02	93,57	7,37E-16	123,18
Gm5089	1,75E-12	233,92	0,14	104,54	5,44E-15	127,90
Tmem47	1,54E-12	233,42	2,49E-07	129,11	3,56E-16	126,88
Ttll12	7,06E-07	232,20	0,01	108,55	2,20E-16	145,75
Caml	1,51E-11	231,77	0,84	99,07	1,13E-14	123,27
Vmn1r43	1,05E-10	230,54	7,30E-08	119,28	2,20E-16	130,60
Aldh16a1	8,22E-15	230,30	3,16E-13	124,11	0,00	107,76
Gjb5	1,37E-06	228,50	0,02	109,01	1,54E-14	134,06
Mum1	4,54E-14	228,00	0,00	109,40	3,20E-16	126,19
Fgf2	3,56E-10	227,57	0,09	102,42	4,81E-13	123,45
Dap3	1,14E-11	227,05	7,17E-06	114,62	0,00	107,35
Cdkal1	7,41E-12	226,30	1,98E-06	88,53	3,30E-08	114,47
Akap10	1,10E-11	226,09	3,08E-07	114,13	2,20E-16	133,61
Dgka	8,92E-14	225,20	0,04	106,02	2,14E-13	121,90
Masp2	3,53E-09	225,18	3,32E-05	111,50	6,02E-07	111,19
Mmgt1	1,18E-10	224,90	1,62E-12	124,16	1,01E-05	110,24
Irf7	4,23E-06	224,54	0,41	104,58	0,00	106,65
Lins	8,51E-10	223,44	0,00	109,12	2,20E-16	132,09
Pdcd6	1,53E-13	223,34	0,10	105,46	1,35E-12	123,57
4933425O20Rik	1,74E-14	221,19	2,09E-15	129,67	2,25E-08	113,93
Cd248	3,97E-12	220,04	3,18E-10	120,37	2,03E-15	127,05
Scube3	6,66E-06	219,78	0,00	108,60	3,66E-14	132,37
Clec4e	1,33E-08	219,18	0,21	103,71	2,20E-16	133,57
Tnfrsf26	8,83E-14	217,91	0,02	106,21	2,20E-16	142,27
Wdr8	9,40E-15	216,91	5,14E-10	117,41	1,01E-15	129,06
Kiss1r	1,33E-13	216,76	0,39	97,76	5,82E-13	119,02
Nudcd2	6,28E-10	216,74	0,05	106,28	0,05	104,80
Gpr135	1,06E-09	216,54	9,46E-05	109,50	5,59E-15	123,38
Tdrd7	2,64E-07	216,27	0,78	99,20	7,64E-15	129,02
Chic1	2,85E-13	215,72	0,00	115,87	2,58E-15	126,13
Tlr8	5,83E-11	215,20	0,00	108,48	1,80E-13	121,98
D19Wsu162e	5,41E-14	214,93	2,87E-10	118,31	1,52E-11	118,83
Bdh2	8,92E-10	212,16	0,00	106,84	7,18E-11	116,74
Hs3st1	1,73E-13	212,02	1,81E-09	119,15	2,24E-13	121,89
Ptgr1	6,29E-12	211,36	3,77E-05	111,21	5,02E-13	121,05
Arx	1,57E-11	210,95	9,47E-05	110,99	6,37E-13	122,32
Cc2d2a	7,03E-14	209,96	1,38E-05	112,50	2,20E-16	130,71
Pcgf3	1,34E-09	209,77	0,62	98,79	1,76E-14	125,62
Chst14	9,16E-09	209,44	0,67	100,04	6,50E-14	123,98
Zfp101	3,49E-13	207,42	0,02	106,45	2,20E-16	131,29
Itgb4	4,30E-11	207,41	0,04	105,38	3,82E-14	121,99
Tmem126a	5,76E-14	206,82	1,30E-06	111,71	2,20E-16	128,10
Maged1	1,21E-12	206,30	0,02	104,59	2,64E-14	120,52
Il1rl2	1,50E-09	206,09	0,57	101,63	1,42E-13	124,01
Ccdc50	3,20E-12	205,41	0,77	101,20	3,14E-15	124,27
Srp9	7,02E-13	203,99	0,12	103,84	2,20E-16	125,95
2510006D16Rik	2,65E-10	203,06	0,23	96,92	1,66E-14	125,41
Plp2	9,83E-12	202,56	1,68E-06	114,97	2,39E-11	119,54
Mael	1,86E-09	202,32	6,35E-05	113,28	1,12E-11	118,24
Chuk	3,35E-10	201,89	0,00	107,80	0,66	100,27
Gapvd1	8,21E-10	201,79	5,18E-06	112,49	4,79E-11	122,60
Mon1a	1,53E-08	201,21	2,56E-09	116,74	0,96	100,43
Fbxo9	1,33E-10	200,50	0,00	109,60	5,06E-06	111,77
Fam136a	2,09E-10	198,72	0,00	110,83	4,29E-07	111,56
Tgfb3	1,17E-10	198,32	0,19	97,46	3,26E-10	117,66
Mmadhc	2,77E-12	198,30	0,02	104,83	2,20E-16	126,17
Nfkbia	9,99E-14	197,96	0,58	100,75	7,18E-15	126,20
Nudt3	1,34E-11	197,40	0,02	105,79	2,20E-16	127,21

Eps15	1,40E-10	196,67	1,09E-06	112,99	4,51E-11	116,98
Ttll9	2,28E-05	194,84	5,88E-10	119,65	0,70	100,69
Stk11	2,58E-09	193,34	0,02	106,17	8,00E-13	121,24
Bbs10	1,89E-08	193,07	6,67E-07	114,47	0,00	92,19
Cnnm4	7,07E-12	192,83	0,15	103,62	1,19E-14	124,09
Pskh1	1,64E-07	192,58	0,17	97,27	8,27E-07	111,37
Dclre1b	6,98E-10	191,54	0,00	91,71	4,17E-16	123,05
4930590J08Rik	1,89E-08	191,52	2,32E-05	90,00	5,51E-05	110,82
Blnk	0,00	191,29	0,00	94,01	5,24E-06	89,09
Mzt1	2,41E-08	190,96	0,07	95,79	6,05E-14	122,68
Slc38a3	3,24E-06	190,90	0,00	108,84	0,01	93,27
Clpb	1,15E-08	190,06	3,25E-06	114,10	1,38E-08	114,71
Myocd	9,99E-12	189,54	0,01	108,59	4,55E-09	116,03
Pram1	7,29E-09	189,41	0,00	110,61	8,20E-13	120,51
Snx16	2,51E-05	188,92	0,90	100,14	2,96E-16	130,89
Cdkn1a	6,44E-09	188,46	4,22E-12	122,56	2,20E-16	62,96
Cfh	1,13E-08	188,34	1,19E-06	89,40	1,91E-10	115,91
Cpeb4	1,10E-10	188,27	0,08	105,39	1,53E-11	116,17
Pak1ip1	1,75E-12	187,84	9,84E-10	116,90	0,00	107,83
Nfatc2	1,47E-11	186,19	2,46E-05	122,86	7,75E-09	132,65
Unc45a	1,68E-10	186,01	0,00	108,44	1,17E-12	79,20
Arhgap23	5,91E-11	185,84	0,04	103,90	1,31E-12	119,98
Rhbdl3	2,29E-09	182,95	0,50	98,85	9,06E-13	127,75
Tgfbr3	1,30E-09	182,53	0,00	106,43	1,26E-12	120,42
Mas1	4,80E-09	182,39	0,45	98,47	2,20E-16	124,31
Camkk2	2,64E-07	181,83	0,08	105,43	0,17	102,34
Hmgcll1	2,00E-07	181,52	0,40	99,65	1,69E-13	125,16
Fabp3	1,01E-09	179,79	1,34E-06	112,38	4,04E-05	109,44
Acp2	1,27E-08	179,65	0,42	102,51	9,37E-10	116,83
Tmem128	4,92E-09	179,07	0,01	93,77	1,05E-07	112,22
Nt5c	2,27E-05	178,19	0,06	95,51	2,09E-10	118,00
Rnf130	2,55E-08	177,38	1,69E-07	88,06	3,68E-11	116,82
Smg6	6,28E-09	177,29	2,63E-15	76,95	0,04	124,29
Aqp9	7,25E-08	177,00	0,73	100,57	7,78E-10	115,65
Cr1l	1,33E-07	174,85	0,00	91,70	0,08	103,29
Rapgef4	1,07E-06	173,99	0,79	98,65	2,34E-10	117,74
Btbd17	9,27E-07	173,71	0,14	103,95	1,03E-07	111,74
Fads1	3,59E-09	173,25	0,53	98,49	0,00	107,46
Idh3a	6,47E-08	173,16	0,98	100,91	2,41E-10	117,84
Muted	3,02E-07	172,42	0,19	96,72	0,08	94,51
Gsto1	8,41E-08	172,27	0,02	107,12	7,54E-07	111,63
Siglece	8,20E-05	172,16	0,13	95,97	5,70E-12	119,72
Atg14	3,45E-06	171,65	0,00	108,54	0,01	106,93
Mknk2	5,19E-05	170,96	1,14E-08	85,80	2,63E-15	124,61
2610301B20Rik	8,37E-07	169,43	0,77	100,59	7,77E-08	111,88
Man2b1	6,93E-09	168,06	9,77E-05	91,26	2,82E-08	113,47
Ptcd1	1,85E-08	167,17	0,51	102,30	0,00	107,68
Fbxl3	8,38E-09	166,61	0,01	107,39	0,25	103,23
Dmrt2	5,63E-08	166,15	0,41	101,54	6,42E-05	90,60
Tmem35	2,22E-08	164,93	0,08	95,77	0,00	107,30
Tex19.1	4,58E-08	164,67	6,23E-08	112,55	1,21E-08	86,72
Arl4c	2,42E-05	164,31	0,09	104,42	1,91E-06	110,43
Map3k2	2,36E-05	164,01	0,80	101,31	0,27	102,75
Iffo2	2,72E-05	163,50	0,04	94,87	0,08	104,63
Drg2	1,83E-07	161,47	0,12	103,72	0,84	99,54
Ak4	2,46E-05	160,36	0,06	105,65	3,24E-11	81,63
Reep1	1,26E-08	159,79	0,06	95,21	0,09	95,82
Mapkapk2	5,41E-06	159,12	0,65	101,06	0,00	92,54
Fbxo18	1,31E-05	157,98	0,24	103,29	0,79	100,80
Atg4c	1,75E-07	157,51	0,15	96,09	0,00	90,41
Olfr986	4,57E-08	157,03	0,00	91,86	0,01	105,07
Calcrl	0,00	154,85	0,02	106,39	0,08	95,27
Tgm2	7,88E-05	154,75	1,15E-07	85,95	9,49E-14	124,90
2610528E23Rik	1,38E-08	153,73	0,00	108,57	8,09E-06	89,84
2700049A03Rik	9,00E-07	152,25	2,45E-12	82,28	3,79E-10	114,43
Zfyve1	1,04E-06	151,14	2,28E-05	111,79	0,02	94,37
Efhc1	9,54E-06	147,46	6,28E-07	88,09	0,88	99,69

Tas2r126	0,00	145,69	1,30E-07	86,36	4,52E-09	85,10
Mthfd2	0,00	145,20	0,15	103,75	0,18	94,83
Myo1e	1,91E-05	141,26	0,77	100,14	0,17	96,14

Supplementary Table 1

Scores (as normalized to 100 to the effect of negative controls, RLuc and FLuc), accompanied with their respective P-values, obtained after esiRNA transfection of the indicated genes into the mouse myogenic cell line C2C12. Effects on fusion (nuclei per fibre, in red), proliferation/cell survival (nuclei count, in blue) and myogenic differentiation (myogenin expression, in green) after loss of function of those molecules are indicated. The list encompasses the gene names (Entrez gene IDs) of the esiRNAs that obtained the highest score in the fusion assay. The corresponding genes are therefore predicted to act as strongest inhibitors of C2C12 fusion.

Table 2. Genes necessary for C2C12 fusion

Entrez Gene ID	pvalue	%nuclei/myotube	pvalue	%nuclei	pvalue	%myogenin
Mrps10	2,20E-16	34,13	1,20E-15	77,32	3,89E-12	76,46
Tfrc	2,20E-16	34,95	2,20E-16	63,29	2,20E-16	69,01
Fau	2,20E-16	38,18	3,15E-16	77,16	7,78E-14	77,76
Psmd6	2,20E-16	39,28	5,92E-16	74,56	2,20E-16	66,09
Snrpd1	2,20E-16	40,39	2,20E-16	74,32	1,58E-11	79,81
Eif2s1	2,20E-16	44,04	3,26E-11	82,37	0,01	92,34
Prpf8	2,20E-16	44,35	2,24E-06	87,23	1,07E-09	85,17
Sdad1	2,20E-16	44,99	2,20E-16	72,94	0,02	94,73
Dcaf13	2,20E-16	45,03	2,48E-14	76,98	9,57E-10	84,08
Mprip	2,13E-15	45,33	5,95E-09	84,22	4,79E-13	77,27
9830001H06Rik	2,20E-16	45,33	0,01	104,89	1,66E-06	90,10
Klhdc3	2,20E-16	46,10	0,67	100,54	1,42E-15	76,26
Hmx1	2,20E-16	46,15	8,88E-08	87,17	3,13E-14	80,67
Sec24a	2,20E-16	46,51	4,22E-08	86,35	3,47E-13	81,26
Atp7b	5,72E-16	46,91	0,01	93,72	3,38E-08	86,39
Hook3	2,07E-15	47,42	1,44E-13	81,99	2,20E-16	72,09
Mapk14	1,10E-14	47,61	2,63E-09	86,96	2,20E-16	71,97
Edf1	1,06E-15	48,02	0,01	107,11	2,20E-16	57,52
Olfr1321	7,73E-15	48,18	7,56E-14	79,04	2,01E-06	87,60
Ubap2l	2,20E-16	48,35	1,04E-11	83,36	6,74E-06	87,17
Zfp513	2,04E-14	48,50	4,44E-12	80,91	3,15E-09	83,70
Tmem40	1,85E-15	48,56	0,86	99,76	3,50E-16	70,35
AA467197	8,38E-15	48,73	0,00	107,42	1,24E-11	82,15
Ubxn10	1,48E-14	48,78	0,00	89,80	0,00	90,70
Gnl3l	1,50E-15	48,95	7,69E-15	73,72	0,00	85,27
Micalcl	2,25E-15	49,06	3,09E-10	82,43	0,00	108,67
Plcg2	7,19E-16	49,09	8,74E-11	83,13	5,45E-13	82,29
Rgmb	2,20E-16	49,23	0,45	101,30	0,46	99,16
Katnb1	1,56E-15	49,23	4,16E-08	86,79	5,76E-06	89,17
Prrc2c	2,18E-13	49,54	2,93E-15	75,56	0,22	96,23
Gsk3b	1,94E-15	49,62	3,13E-06	88,71	6,85E-14	77,28
Asap1	3,32E-14	50,45	1,12E-08	85,22	4,55E-15	77,80
Dhx30	1,48E-14	50,61	2,30E-08	84,81	0,00	107,52
Pknox1	4,13E-15	50,90	9,86E-14	78,18	0,00	90,17
Blm	1,38E-13	50,91	1,37E-06	87,50	3,83E-12	79,99
Bnip2	4,29E-14	51,39	5,46E-08	85,91	3,93E-05	89,26
Exoc6b	4,75E-13	52,05	0,02	106,49	2,07E-06	89,12
Plekhf1	1,83E-12	52,39	9,24E-09	84,48	4,64E-11	79,86
Catsper2	4,96E-14	52,48	0,01	93,97	0,64	98,39
Dnalc4	1,24E-14	52,79	1,88E-06	89,89	1,28E-07	88,26
Lingo2	3,43E-13	53,13	4,18E-07	87,23	0,00	91,84
C1ql3	1,33E-12	53,19	6,11E-10	83,84	0,00	88,93
Eci2	4,98E-13	53,24	0,40	101,42	0,50	99,18
Atp6ap1	5,08E-13	53,75	9,39E-10	84,93	2,39E-07	113,69
Arhgap30	1,49E-13	53,79	0,77	98,65	3,90E-07	113,62
Crls1	5,77E-14	54,44	2,82E-05	110,84	1,10E-07	114,62
Brca2	2,60E-12	54,49	3,92E-11	83,04	0,00	92,61
Melk	2,23E-14	54,69	0,04	95,16	8,23E-09	86,99
Eci1	2,90E-14	55,44	0,30	97,41	1,06E-08	112,49
Olfr1040	1,82E-12	55,71	2,18E-09	85,89	3,00E-05	88,05
Amz1	4,71E-12	55,96	0,07	95,25	2,23E-07	88,52
Tmem87a	1,03E-12	56,51	2,23E-14	77,29	0,00	93,07
Prkca	2,61E-13	56,62	1,22E-09	84,68	2,80E-09	86,59
Atp6v0e	1,55E-11	56,92	4,11E-08	87,00	1,97E-06	89,53
Sema5b	5,62E-12	57,00	3,22E-10	84,15	2,86E-10	84,13
Srsf1	7,37E-12	57,25	1,72E-10	83,92	0,02	106,10
Arhgap17	2,63E-13	57,38	0,00	92,92	0,01	105,00
Jarid2	1,57E-13	57,40	0,37	97,24	8,98E-10	83,56
Tmem186	4,23E-11	57,54	0,02	106,17	0,00	106,47
Trim45	3,97E-12	57,64	2,97E-09	85,88	0,88	99,66
Idua	3,27E-10	57,85	2,56E-11	80,31	3,41E-14	124,50
1700001O22Rik	4,91E-09	58,19	0,97	100,94	0,00	88,76
Gria4	5,67E-10	58,21	0,24	97,70	1,06E-14	75,38

Cct8l1	3,11E-11	58,38	1,75E-08	84,59	5,27E-09	86,07
Triobp	2,37E-12	59,10	6,77E-07	89,98	1,66E-13	81,21
1110008J03Rik	1,14E-11	59,46	0,02	93,29	2,78E-13	78,42
Rinl	1,08E-11	59,63	0,00	91,35	8,60E-07	89,17
Specc1l	5,92E-11	59,87	0,11	104,26	0,93	99,75
Pkd1l2	4,30E-10	60,41	1,87E-08	84,77	0,41	101,97
Taf15	3,04E-11	61,06	0,00	91,21	0,17	97,18
Chmp4c	2,89E-09	61,11	0,07	105,50	1,00E-06	88,23
Gpr30	9,29E-11	61,23	0,11	96,80	6,05E-12	81,99
Zfp697	3,92E-10	61,33	7,61E-06	88,81	5,54E-06	86,81
Smpd4	1,02E-10	61,70	0,01	106,34	0,00	94,52
Creb3l4	2,83E-10	61,71	0,49	101,58	4,35E-12	80,25
Cep97	1,21E-10	62,00	1,14E-06	88,62	8,24E-06	89,03
Slc39a1	5,36E-10	62,04	0,09	95,26	0,17	97,23
Sall4	2,28E-09	62,05	0,29	95,87	6,24E-13	80,52
Ruvbl2	1,67E-09	62,20	0,46	98,00	6,21E-07	88,61
Haus8	2,53E-09	62,21	0,53	100,78	0,00	92,19
Lrmp	3,37E-09	62,21	0,05	95,52	9,58E-09	85,86
Dnajc24	2,39E-10	62,22	2,22E-09	85,50	0,01	94,89
Psrc1	3,95E-10	62,40	1,15E-06	89,58	0,61	101,64
Rgs5	2,05E-10	62,65	0,00	110,90	0,00	92,95
Nlgn2	4,48E-10	62,78	0,00	92,14	0,00	92,54
Hoxc5	9,63E-10	63,07	0,47	97,52	6,44E-12	82,57
Ezr	5,81E-09	63,19	0,02	94,03	2,45E-05	88,73
Fktn	3,35E-09	63,19	4,51E-06	87,41	0,66	100,94
Kif3c	1,58E-09	63,26	0,06	95,91	9,46E-11	84,46
Ncoa2	1,95E-09	63,33	0,00	92,57	2,22E-08	86,14
Wwtr1	1,39E-10	63,47	0,01	92,94	6,57E-14	120,73
Hmbs	2,53E-09	63,71	8,05E-14	127,11	2,58E-05	90,81
Zdhhc25	3,48E-09	63,74	0,00	106,43	1,27E-06	111,36
Fam125a	6,60E-10	63,77	9,67E-08	111,96	2,76E-12	81,19
Bag3	7,84E-10	63,85	0,00	107,11	6,55E-07	88,63
Itgae	2,18E-09	64,04	0,00	108,79	7,97E-07	88,10
Mepce	2,16E-08	64,05	0,10	103,55	2,49E-06	110,18
Irak1	8,26E-10	64,19	2,22E-10	118,00	1,55E-11	84,00
Tspan7	1,19E-09	64,24	0,54	98,36	0,13	97,09
Rcsd1	3,52E-09	64,31	0,31	97,48	4,71E-07	112,66
Dennd4b	1,82E-08	64,43	0,00	92,78	0,71	101,01
Idh1	7,06E-09	64,76	3,99E-05	89,52	0,01	104,90
Psmc5	2,51E-08	64,78	0,56	101,33	0,00	91,91
Zfp341	3,49E-09	64,79	0,00	91,49	0,01	94,48
Dnajb5	1,81E-08	65,18	0,35	97,96	0,01	94,72
Fgf3	8,80E-09	65,32	5,90E-09	120,01	0,01	105,52
Rab39	5,48E-08	65,33	0,39	101,87	0,01	93,02
Tcf15	3,37E-09	65,46	0,00	108,27	1,60E-06	87,42
Ppp2r1a	2,70E-08	65,56	0,08	95,76	3,12E-05	90,72
Cnot6l	9,02E-08	65,57	0,22	102,16	0,02	95,61
Gm2a	2,54E-08	65,61	0,58	98,74	0,00	91,82
Srrm2	2,30E-08	65,70	0,87	99,24	0,02	94,13
Tinf2	2,81E-08	65,89	0,02	94,45	0,23	102,17
Cldn13	1,31E-08	65,95	3,35E-10	121,81	4,20E-06	109,84
1810037I17Rik	3,54E-08	66,07	0,13	96,56	8,33E-09	85,14
Syt7	3,68E-09	66,16	0,00	105,22	0,00	92,93
Vta1	5,68E-08	66,18	0,09	96,15	0,00	92,64
Reep2	1,71E-07	66,27	0,00	91,91	0,01	94,36
Galntl6	1,87E-07	66,38	3,53E-12	80,75	0,01	105,62
Rhod	1,35E-07	66,42	0,00	93,24	0,00	108,82
Cpsf1	6,47E-08	66,54	1,26E-05	111,04	0,04	96,26
Zbtb46	1,65E-08	66,81	4,57E-09	86,19	0,56	100,76
Apba1	9,07E-08	66,86	0,02	95,05	0,37	103,25
Syt5	2,79E-08	66,95	0,04	95,06	0,03	94,98
Grpel1	1,45E-07	67,09	0,35	101,64	3,76E-06	89,89
Metap2	8,05E-07	67,12	0,03	92,58	0,09	95,98
Cenpb	6,87E-08	67,12	0,03	95,33	0,22	97,58
Bcor	4,40E-08	67,15	5,77E-07	88,41	0,31	98,01

Usp29	1,17E-07	67,68	0,09	96,18	0,00	108,15
Treml1	4,17E-07	67,82	0,26	102,26	0,56	98,89
Fxyd5	1,10E-07	68,03	4,03E-05	89,70	0,15	96,18
Kcnh3	7,05E-07	68,05	0,00	91,25	0,12	97,07
Aoc3	1,76E-07	68,06	0,00	109,96	8,57E-07	113,09
Fhl1	2,03E-06	68,09	0,00	110,02	2,01E-08	84,38
Ncapd3	3,25E-07	68,45	0,79	98,62	2,25E-07	113,36
Plat	2,39E-06	68,49	0,98	100,83	6,62E-05	112,13
Slc26a11	1,68E-06	68,61	0,07	107,08	0,01	106,09
Stxbp1	4,36E-07	68,62	0,00	108,28	0,00	92,18
Hook2	8,68E-07	68,77	2,20E-16	143,06	0,65	101,04
6030419C18Rik	3,82E-07	69,00	0,74	98,46	0,01	93,50
Cdh15	6,29E-07	69,01	0,00	107,96	0,42	101,93
Pi4k2b	2,32E-07	69,22	0,45	98,22	1,19E-07	111,81
Skor1	6,04E-07	69,23	0,61	101,54	0,01	93,59
Runx1t1	3,13E-06	69,47	0,97	99,53	4,39E-05	91,55
Rin1	8,89E-07	70,21	0,24	96,03	3,23E-15	125,35
Ccdc104	1,29E-05	70,33	8,52E-05	88,70	3,35E-06	114,59
Fgf15	3,60E-06	70,93	0,69	100,46	2,12E-05	89,07
Col8a1	7,17E-06	70,96	0,14	95,64	0,78	100,54
Plk2	4,31E-06	71,02	0,00	92,16	0,11	103,68
Ppt2	2,98E-06	71,04	0,00	93,22	0,00	108,02
Mcam	2,34E-06	71,08	4,20E-06	110,29	0,00	93,40
Naca	1,42E-05	71,12	0,24	103,05	0,00	89,68
Chchd7	6,73E-06	71,20	3,41E-06	89,78	0,48	97,96
Egfr	3,54E-06	71,21	0,46	98,36	0,01	105,14
Cxcr7	2,46E-06	71,58	0,44	101,16	0,00	105,50
A2m	9,62E-06	71,69	0,04	95,46	0,00	106,53
Gja4	3,88E-05	71,81	0,75	103,28	2,01E-05	89,98
Fam111a	2,25E-05	71,87	0,48	97,97	0,83	100,31
Syp	3,83E-06	71,89	0,25	103,19	3,43E-13	119,78
Stag2	5,85E-06	72,02	4,21E-09	85,46	0,14	103,32
Psmc2	3,09E-05	72,05	0,03	96,53	0,00	93,66
8430408G22Rik	1,60E-05	72,08	0,14	103,00	0,15	96,73
2310043J07Rik	5,77E-06	72,14	0,00	92,20	0,60	101,36
Ror2	3,20E-05	72,23	0,00	109,23	0,00	93,19
Acads	6,88E-05	72,25	0,00	91,41	0,00	109,84
Armcx6	3,49E-05	72,30	0,66	99,13	0,03	105,83
Nif3l1	0,00	72,52	1,35E-05	89,40	0,02	100,01
Mpg	9,30E-06	72,61	0,01	94,91	0,00	108,37
Dido1	3,33E-05	72,69	0,23	103,46	7,03E-05	107,87
Nckap1	7,67E-06	72,70	0,23	97,18	2,20E-16	130,30
Klc4	1,60E-05	72,86	0,02	104,36	3,72E-05	87,60
Gata4	1,24E-05	73,14	0,18	102,49	0,01	93,53
Akr1c12	0,00	73,39	3,58E-05	90,11	0,01	105,24
Marveld2	1,66E-05	73,40	1,33E-11	125,18	2,20E-16	138,46
Aldoc	5,43E-05	73,42	0,52	101,75	0,65	101,06
Kank2	6,92E-05	73,70	0,05	105,10	5,52E-07	86,51
Il4ra	2,30E-07	73,74	0,00	106,98	0,04	96,08
Col6a2	4,23E-05	73,75	0,00	110,30	2,51E-08	87,24
Arhgef7	5,11E-05	73,80	0,01	93,60	0,08	103,46
Ap2m1	6,34E-05	73,83	0,09	96,28	1,69E-07	115,15
Fbl	9,77E-05	73,84	0,05	95,59	0,00	107,64
Cdc25b	0,00	73,92	0,77	103,15	7,43E-07	88,43
Fus	4,89E-05	74,05	0,00	106,34	1,25E-06	109,31
Ccl2	7,51E-06	74,51	0,25	96,21	0,95	99,76
Exoc3l	0,00	74,92	3,35E-05	109,53	0,76	98,97
Rnf123	7,14E-05	75,05	0,64	100,97	4,26E-09	114,08
Rcc2	0,00	75,34	1,66E-10	117,21	0,05	96,34
Sphk1	0,00	75,56	9,54E-10	117,15	2,19E-09	86,44
Lrtm2	0,00	75,62	0,41	101,97	0,47	102,04
Scp2	0,00	76,24	9,34E-16	76,99	3,53E-14	121,93
Rtn4ip1	0,00	76,30	0,84	100,23	0,80	100,70
Dicer1	0,00	76,49	0,99	99,40	3,12E-08	111,89
Arap3	0,00	76,54	0,00	93,38	0,00	106,59

Tmem63a	0,00	76,61	0,97	99,30	0,88	99,14
Scaf1	0,00	77,13	0,01	108,58	0,00	105,95
Zfp105	0,00	77,29	0,28	102,98	1,93E-06	112,93
Rtn2	0,00	77,31	0,26	95,97	0,00	108,61
Pdcd4	0,00	77,49	0,01	106,45	4,05E-08	113,10
Sox15	0,00	77,58	0,00	92,23	0,02	104,41
Fermt3	0,00	79,35	7,73E-05	111,41	0,02	104,79
4930563D23Rik	0,00	82,53	0,00	93,65	4,47E-09	118,02

Supplementary Table 2

Scores (as normalized to 100 to the effect of negative controls, RLuc and FLuc), accompanied with their respective P-values, obtained after esiRNA transfection of the indicated genes into the mouse myogenic cell line C2C12. Effects on fusion (nuclei per fibre, in red), proliferation/cell survival (nuclei count, in blue) and myogenic differentiation (myogenin expression, in green) after loss of function of those molecules are indicated. The list encompasses the Entrez gene IDs of the esiRNAs that obtained the lowest score in the fusion assay. The corresponding genes are therefore predicted to act as most necessary for C2C12 fusion.

Table 3. Known fusion genes (with references) tested in the esiRNA screen

Gene	*Modulate C2C12 fusion (esiRNA test)*	*References*	*Remark*
Anxa1	No	Leikina et al. J Cell Biol 2013 [1]	Anxa1 & a5 act redundantly
Anxa5	No	Leikina et al. J Cell Biol 2013 [1]	Anxa1 & a5 act redundantly
Arhgap17	Yes	Doherty et al. J Biol Chem 2011 [2]	
Arhgap23	Yes	Doherty et al. J Biol Chem 2011 [2]	
Arhgap30	Yes	Doherty et al. J Biol Chem 2011 [2]	
Bai3	No	Hamoud et al. Nature Comm 2018 [3]	
Capn7	Yes	Buffolo et al. Genesis 2015 [4]	
Cdc42	Yes	Vasyutina et al. PNAS 2009 [5]	
Cdh15/m-Cadh	Yes	Charrasse et al. Mol Cell Biol 2007 [6]	
Ciql3	Yes	Hamoud et al. Nature Comm 2018 [3]	
Dock1	NT	Laurin et al. PNAS 2008 [7]	
Fhl1	Yes	Cowling et al. J Cell Biol 2008 [8]	
Graf1	NT	Lenhart et al. Dev Biol 2014 [9]	
Il4ra	Yes	Horsley et al. Cell 2003 [10]	
Itga6	Yes	Wilschut et al. Stem Cell Research 2011 [11]	
Itgb1	NT	Schwander et al. Dev Cell 2003 [12]	
Jam2	No	Powell and Wright Plos Biol 2011 [13]	Jam2 & 3 act redundantly
Jam3	NT	Powell and Wright Plos Biol 2011 [13]	Jam2 & 3 act redundantly
Mapk7/Erk5	NT	Sunadome et al. Dev Cell 2011 [14]	
Myd88	Yes	Hindi et al. Nature Comm 2017 [15]	
Mymk	NT	Millay et al. Nature 2013 [16]	
Mymx	NT	Quinn et al. Nature Comm 2017; Bi et al. Science 2017; Zhang et al. Nature Comm 2017 [17,18,19]	
Myof	No	Doherty et al. Development 2005 [20]	
Neogenin	NT	Bae et al. Mol Biol Cell 2009 [21]	
Nfatc2	Yes	Horsley et al. Cell 2003 [10]	
Nphs1/Nephrin	NT	Sohn et al. PNAS 2009 [22]	
Npnt	Yes	Lee et al. Sci Rep 2017 [23]	
Nr2f2/Coup TFII	Yes	Lee et al. Sci Rep 2017 [23]	
Pak1	Yes	Duan et al. J Cell Biol 2012 [24]	
Prkcq	Yes	Madaro et al. Mol Biol Cell 2011 [25]	
Ptk2/Fak	NT	Quach et al. Mol Biol Cell 2009 [26]	
Rac1	Yes	Vasyutina et al. PNAS 2009 [5]	
RhoA	NT	Wei et al. JBC, 1998 [27]	
Stab2	No	Park et al. Nature Comm 2016 [28]	
Wasl/N-Wasp	Yes	Gruenbaum-Cohen et al. PNAS 2012 [29]	

No: No effect on C2C12 fusion
Yes: Effect on C2C12 fusion
NT: Not tested

Supplementary Table 3
List of genes known from the literature to modulate fusion (with references) and that were tested or not in the esiRNA screen in the present study.

Table 3. Role of TGFβ-associated genes on C2C12 fusion

TGFβ ligands (TGFBs, INHs, MST, NODAL)	Fusion (% of Ctrl)	Proliferation	Differentiation (MyoG)
TGFB1	ns		
TGFB2	406	112	154
TGFB3	198	97	118
INHA	ns		
INHBC	ns		
INHBE	ns		
FST (Myostatin inhibitor)	ns		
TGFβ receptor			
TGFBR1	284	97	149
TGFBR3 (co-receptor)	183	106	120
ACVR1B	254	112	124
ACVRL1	ns		
ACVR2B (MST unique receptor II)	ns		
ACVR2A	ns		
Effectors			
SMAD2	166	91	131
SMAD3	378	100	137
SMAD4	524	98	157
Synergizes with TGFβ(ref)			
DPT(30)	148	113	113
MMP14(31)	154	90	126
RUNX1(32)	179	107	125
SCUBE3(33)	220	108	132
TGFβ antagonists			
TGIF1	57	88	79
BMP pathway			
BMPR1A	ns		
BMP 1, 4, 7, 8	ns		
ACVR2A, 2B	ns		
SMAD5	ns		
Positive controls			
RAC1	73	85	142
CDC42	52	81	140

ns: non significant

note: TGFB1,2,3 bind TGFBR1 or ACVRL1 (type I receptors) and only bind TGFBR2 (type II receptors)

MST binds TGFBR1 or ACVR1B (type I receptors) and only binds ACVR2B (type II receptor)

References for Supplementary Information

1. Leikina, E. *et al.* Extracellular annexins and dynamin are important for sequential steps in myoblast fusion. *J. Cell Biol.* (2013). doi:10.1083/jcb.201207012
2. Doherty, J. T. *et al.* Skeletal muscle differentiation and fusion are regulated by the BAR-containing Rho-GTPase-activating Protein (Rho-GAP), GRAF. *J. Biol. Chem.* (2011). doi:10.1074/jbc.M111.243030
3. Hamoud, N. *et al.* Spatiotemporal regulation of the GPCR activity of BAI3 by C1qL4 and Stabilin-2 controls myoblast fusion. *Nat. Commun.* (2018). doi:10.1038/s41467-018-06897-5
4. Buffolo, M., Batista Possidonio, A. C., Mermelstein, C. & Araujo, H. A conserved role for calpains during myoblast fusion. *Genesis* (2015). doi:10.1002/dvg.22870
5. Vasyutina, E., Martarelli, B., Brakebusch, C., Wende, H. & Birchmeier, C. The small G-proteins Rac1 and Cdc42 are essential for myoblast fusion in the mouse. *Proc. Natl. Acad. Sci. U. S. A.* **106**, 8935–40 (2009).
6. Charrasse, S. *et al.* M-cadherin activates Rac1 GTPase through the Rho-GEF Trio during myoblast fusion. *Mol. Biol. Cell* (2007). doi:10.1091/mbc.E06-08-0766
7. Laurin, M. *et al.* The atypical Rac activator Dock180 (Dock1) regulates myoblast fusion in vivo. *Proc. Natl. Acad. Sci. U. S. A.* (2008). doi:10.1073/pnas.0805546105
8. Cowling, B. S. *et al.* Identifi cation of FHL1 as a regulator of skeletal muscle mass: Implications for human myopathy. *J. Cell Biol.* (2008). doi:10.1083/jcb.200804077
9. Lenhart, K. C. *et al.* GRAF1 promotes ferlin-dependent myoblast fusion. *Dev. Biol.* (2014). doi:10.1016/j.ydbio.2014.06.025
10. Horsley, V., Jansen, K. M., Mills, S. T. & Pavlath, G. K. IL-4 acts as a myoblast recruitment factor during mammalian muscle growth. *Cell* (2003). doi:10.1016/S0092-8674(03)00319-2
11. Wilschut, K. J., van Tol, H. T. A., Arkesteijn, G. J. A., Haagsman, H. P. & Roelen, B. A. J. Alpha 6 integrin is important for myogenic stem cell differentiation. *Stem Cell Res.* (2011). doi:10.1016/j.scr.2011.05.001
12. Schwander, M. *et al.* β1 integrins regulate myoblast fusion and sarcomere assembly. *Dev. Cell* (2003). doi:10.1016/S1534-5807(03)00118-7
13. Powell, G. T. & Wright, G. J. Jamb and jamc are essential for vertebrate myocyte fusion. *PLoS Biol.* (2011). doi:10.1371/journal.pbio.1001216
14. Sunadome, K. *et al.* ERK5 Regulates Muscle Cell Fusion through Klf Transcription Factors. *Dev. Cell* (2011). doi:10.1016/j.devcel.2010.12.005
15. Hindi, S. M. *et al.* MyD88 promotes myoblast fusion in a cell-autonomous manner. *Nat. Commun.* (2017). doi:10.1038/s41467-017-01866-w
16. Millay, D. P. *et al.* Myomaker is a membrane activator of myoblast fusion and muscle formation. *Nature* **499**, 301–305 (2013).
17. Quinn, M. E. *et al.* Myomerger induces fusion of non-fusogenic cells and is required for skeletal muscle development. *Nat. Commun.* **8**, (2017).
18. Bi, P. *et al.* Control of muscle formation by the fusogenic micropeptide myomixer. *Science (80-.).* **356**, 323–327 (2017).
19. Zhang, Q. *et al.* The microprotein Minion controls cell fusion and muscle formation. *Nat. Commun.* **8**, (2017).
20. Doherty, K. R. *et al.* Normal myoblast fusion requires myoferlin. *Development* (2005). doi:10.1242/dev.02155
21. Bae, G. U. *et al.* Neogenin regulates skeletal myofiber size and focal adhesion kinase and extracellular signal-regulated kinase activities in vivo and in vitro. *Mol. Biol. Cell* (2009). doi:10.1091/mbc.E09-06-0491
22. Sohn, R. L. *et al.* A role for nephrin, a renal protein, in vertebrate skeletal muscle cell fusion. *Proc. Natl. Acad. Sci. U. S. A.* (2009). doi:10.1073/pnas.0904398106
23. Lee, C. T. *et al.* The Nuclear Orphan Receptor COUP-TFII Is Required for Limb and Skeletal Muscle Development. *Mol. Cell. Biol.* (2004). doi:10.1128/mcb.24.24.10835-10843.2004
24. Duan, R. *et al.* Group I PAKs function downstream of Rac to promote podosome invasion during myoblast fusion in vivo. *J. Cell Biol.* (2012). doi:10.1083/jcb.201204065
25. Madaro, L. *et al.* PKCθ signaling is required for myoblast fusion by regulating the expression of caveolin-3 and β1D integrin upstream focal adhesion kinase. *Mol. Biol. Cell* (2011). doi:10.1091/mbc.E10-10-0821
26. Quach, N. L., Biressi, S., Reichardt, L. F., Keller, C. & Rando, T. A. Focal adhesion kinase signaling

regulates the expression of caveolin 3 and β1 integrin, genes essential for normal myoblast fusion. *Mol. Biol. Cell* (2009). doi:10.1091/mbc.E09-02-0175
27. Wei, L. *et al.* RhoA signaling via serum response factor plays an obligatory role in myogenic differentiation. *J. Biol. Chem.* (1998). doi:10.1074/jbc.273.46.30287
28. Park, S. Y. *et al.* Stabilin-2 modulates the efficiency of myoblast fusion during myogenic differentiation and muscle regeneration. *Nat. Commun.* (2016). doi:10.1038/ncomms10871
29. Gruenbaum-Cohen, Y. *et al.* The actin regulator N-WASp is required for muscle-cell fusion in mice. *Proc. Natl. Acad. Sci. U. S. A.* (2012). doi:10.1073/pnas.1116065109
30. OKAMOTO, O., FUJIWARA, S., ABE, M. & SATO, Y. Dermatopontin interacts with transforming growth factor β and enhances its biological activity. *Biochem. J.* (1999). doi:10.1042/0264-6021:3370537
31. Sounni, N. E. *et al.* Stromal regulation of vessel stability by MMP14 and TGF . *Dis. Model. Mech.* (2010). doi:10.1242/dmm.003863
32. Zaidi, S. K. *et al.* Integration of Runx and Smad regulatory signals at transcriptionally active subnuclear sites. *Proc. Natl. Acad. Sci. U. S. A.* (2002). doi:10.1073/pnas.112664499
33. Wu, Y.-Y. *et al.* SCUBE3 is an endogenous TGF-β receptor ligand and regulates the epithelial-mesenchymal transition in lung cancer. *Oncogene* (2011). doi:10.1038/onc.2011.85
